# A 4‐gene expression score associated with high levels of *Wilms Tumor‐1 (WT1)* expression is an adverse prognostic factor in acute myeloid leukaemia

**DOI:** 10.1111/bjh.13836

**Published:** 2015-11-24

**Authors:** Ahmadreza Niavarani, Tobias Herold, Yasmin Reyal, Maria C. Sauerland, Thomas Buchner, Wolfgang Hiddemann, Stefan K. Bohlander, Peter J. M. Valk, Dominique Bonnet

**Affiliations:** ^1^ Digestive Oncology Research CenterDigestive Disease Research Institute (DDRI)Shariati HospitalTehran University of Medical SciencesTehranIran; ^2^ Haematopoietic Stem Cell LaboratoryLondon Research InstituteCancer Research UKLondonUnited Kingdom; ^3^ Department of Internal Medicine 3University Hospital GrosshadernLudwig‐Maximilians‐UniversitätMunichGermany; ^4^ Department of HaematologyUniversity College London Hospitals NHS TrustLondonUK; ^5^ Institute of Biostatistics and Clinical ResearchUniversity of MünsterMünsterGermany; ^6^ Department of Medicine A ‐ Haematology, Oncology and PneumologyUniversity of MünsterMünsterGermany; ^7^ Department of Molecular Medicine and PathologyThe University of AucklandAucklandNew Zealand; ^8^ Department of HaematologyErasmus University Medical Centre Cancer InstituteRotterdamthe Netherlands

**Keywords:** *WT1*, gene signature, expression score, AML, prognosis

## Abstract

*Wilms Tumor‐1 (WT1)* expression level is implicated in the prognosis of acute myeloid leukaemia (AML). We hypothesized that a gene expression profile associated with *WT1* expression levels might be a good surrogate marker. We identified high *WT1* gene sets by comparing the gene expression profiles in the highest and lowest quartiles of *WT1* expression in two large AML studies. Two high *WT1* gene sets were found to be highly correlated in terms of the altered genes and expression profiles. We identified a 17‐probe set signature of the high *WT1* set as the optimal prognostic predictor in the first AML set, and showed that it was able to predict prognosis in the second AML series after adjustment for European LeukaemiaNet genetic groups. The gene signature also proved to be of prognostic value in a third AML series of 163 samples assessed by RNA sequencing, demonstrating its cross‐platform consistency**.** This led us to derive a 4‐gene expression score, which faithfully predicted adverse outcome. In conclusion, a short gene signature associated with high *WT1* expression levels and the resultant 4‐gene expression score were found to be predictive of adverse prognosis in AML. This study provides new clues to the molecular pathways underlying high *WT1* states in leukaemia.

Acute myeloid leukaemia (AML) is a heterogeneous disease with variable prognosis depending mainly on the underlying genetic aberrations. AML patients are classified according to different risk‐stratification guidelines, including those of the World Health Organization (WHO) (Swerdlow *et al*, 2008) and the European LeukaemiaNet (ELN) (Dohner *et al*, 2010). These guidelines are mainly based on the presence or absence of specific cytogenetic aberrations and gene mutations. However, the guidelines have evolved over the years, as more and more new factors are found to affect AML prognosis given our increasing knowledge of the AML biology as well as the emergence of modern powerful high‐throughput tools, including gene expression profiling (GEP) and next generation sequencing. Several studies have since attempted to explore the correlation of biologically relevant events to AML‐GEP and prognosis using supervised cluster analysis. These studies have led to identification of several prognostic gene signatures related to various biological or clinical characteristics, including gene rearrangements (Camos *et al*, 2006; Wilson *et al*, 2006), gene mutations in *NPM1* (Verhaak *et al*, 2005), *CEBPA* (Wouters *et al*, 2009), *FLT3* (Neben *et al*, 2005) and *WT1* (Becker *et al*, 2010), gene expression (de Jonge *et al*, 2010), leukaemic stem cells (Gentles *et al*, 2010) and drug sensitivity (Tagliafico *et al*, 2006).

High levels of *WT1* expression were originally found to be associated with poor prognosis in adult AML patients and used as a marker for the detection of minimal residual disease in AML (Inoue *et al*, 1994), as well as in acute lymphoblastic (ALL) (Inoue *et al*, 1994) and chronic myeloid leukaemia (CML) (Inoue *et al*, 1996). The predictive value of isolated high *WT1* expression in AML has been confirmed in several follow‐up long‐term studies (Bergmann *et al*, 1997; Trka *et al*, 2002), and it was in fact extended to therapy subgroups, including haematopoietic stem cell transplantation (Jacobsohn *et al*, 2009), though it was not a consistent finding in this setting (Ostergaard *et al*, 2004). However, despite numerous clinical studies providing solid evidence for the role of high *WT1* levels in leukaemia, its role is not yet clearly defined in the context of other known risk factors relevant for AML prognosis. Moreover, little is known about the molecular alterations associated to high *WT1* levels that can be responsible for its poor prognostic impact. As a transcriptional regulator, WT1 binds to some common DNA binding sites (Rauscher *et al*, 1990), and it is not surprising that changes in its expression levels are associated with changes in the expression of hundreds of genes (Kim *et al*, 2009; Vidovic *et al*, 2013). We hypothesized that high *WT1* expression was the sign of a true biological entity associated with a characteristic gene expression profile, and potentially correlated to AML prognosis. We tested this hypothesis by exploring the GEP differences among high‐ and low‐expressing *WT1* samples in two large AML series and next attempted to predict AML outcome using a gene signature and a gene expression score determined from high *WT1* expression. This can shed some light on the molecular mechanisms underlying the role of high *WT1* in AML pathogenesis as well as prognosis.

## Methods and materials

### Patients and samples

The first series of AML patients, hereafter called ‘Netherlands series’, comprised 524 younger adult (≤60 years) cases who have been treated according to sequential the Dutch‐Belgian Haemato‐Oncology Cooperative Group and the Swiss Group for Clinical Cancer Research (HOVON/SAKK) AML‐04, ‐04A, ‐29, ‐32, ‐42, and ‐43 protocols (Valk *et al*, 2004; de Jonge *et al*, 2010). The second series, hereafter called ‘Germany series’, consisted of 562 adult AML patients who were enrolled in the German AMLCG‐99, AMLCG‐M3, AMLSG AML HD98A or HD98B trial protocols (Herold *et al*, 2014). The third series, hereafter called The Cancer Genome Atlas series (‘TCGA series’), consisted of 163 adult AML patients enrolled in Cancer and Leukemia Group B (CALGB) treatment protocols 8525, 8923, 9621, 9720, 10201, and 19808 including those with survival and immunophenotyping data (The Cancer Genome Atlas Research Network, 2013). The Netherlands (GSE14468) and Germany (GSE37642) gene expression sets were retrieved from NCBI Gene Expression Omnibus (GEO), and the normalized RSEM (RNS sequencing [RNA‐Seq] by Expectation‐Maximization) and clinical AML data set (LAML) were obtained from the TCGA Data Portal (https://tcga-data.nci.nih.gov/tcga/tcgaDownload.jsp).

### Gene expression profiling

The Netherlands Study employed HG‐U133 Plus 2·0 arrays (Affymetrix, Santa Clara, CA, USA) for gene expression profiling (Verhaak *et al*, 2009), while the Germany Study used either HG‐U133 Plus 2·0 or HG‐U133A and U133B arrays (Affymetrix) (Li *et al*, 2013). For practical reasons, only common probe‐sets among two Germany subgroups were used in this study. The normalized RSEM data obtained from RNA‐seq was used as an estimate of TCGA gene expression profiles.

### High *WT1* gene set

Each data series was separately sorted based on *WT1* expression (using probe‐set 206067_s_at), resulting in four quartiles. The fourth quartile (Q4) of the samples with highest mean *WT1* expression was compared with the first quartile (Q1) with lowest mean *WT1* expression. Those probe‐sets with significant differential expression in Q4 as compared to Q1 were considered as the high *WT1* gene set for that AML series.

### Statistical analysis

The two‐tailed student's *t*‐test (IBM spss, v.20, IBM, Armonk, NY, USA) was used to compare the means among Q4 *versus* Q1 samples, with acceptable Benjamini false discovery rate (FDR) of less than 0·05. Linear regression analysis (IBM spss, v.20) was performed to study the correlation of the differences among various gene sets. Overall survival (OS) was measured from the date of patient enrolment to the date of death. Event‐free survival (EFS) was measured from the date of patient enrolment to the date of failure to achieve complete remission, relapse from complete remission or death. Relapse‐free survival (RFS) was measured from the date of complete remission to the date of relapse or death. Cox regression test (IBM SPSS, v.20) was used for univariate and multivariate analysis of survival in the classifier and validation AML series. Univariate Cox regression analysis was performed using each of the potentially implicated variables. Multivariate Cox regression analysis was performed using all variables with *P* < 0·1 in univariate analyses. Kaplan–Meier analysis was performed using Mantel‐Cox statistic (IBM spss, v.20) in order to test the equality of survival distributions for the different levels of the classifier in both the classifier and validation AML series.

### Outcome prediction

The online KNNXValidation tool (version 6) from GenePattern suite (version 3·8·1; Broad Institute, Cambridge, MA, USA) (Reich *et al*, 2006) was employed to predict the prognostic gene signature, with EFS as the favourable event, and all others (dead, no remission, progressive disease/relapse) as adverse events. The Student's *t*‐test (median) was used to select the probe‐sets. The resultant predicting list was trimmed so that only those probe‐sets that repeated in at least 90% of the permutations were kept. GENE‐E (http://www.broadinstitute.org/cancer/software/GENE-E/) was used for supervised clustering of each AML series using trimmed probe‐set list or candidate gene signature.

### Gene expression score

Receiver operating characteristic (ROC) curve analysis (IBM SPSS, v.20) was performed for individual probe‐sets of the gene signature in order to assess their correlations to the EFS in the Netherlands series. Expression scores were developed for each sample using addition of the expression levels for the top two significant probe‐sets considering their positive or negative correlation to the EFS. This was repeated after inclusion of the progressively less significant probe‐sets, giving rise to expression scores for the lists of 2–17 probe‐sets. The median expression scores were used to classify the samples into two groups, and their association to the EFS were assessed using ROC curve analysis.

### Pathway analysis

Pathway analysis was performed using GeneGo MetaCore (https://portal.genego.com). In brief, the gene set was uploaded to MetaCore and Functional Ontology Analysis was performed to identify the enriched pathway maps. Build Networks Analysis was conducted to identify the Shortest Paths among input genes.

## Results

### A high degree of correlation was found between the high *WT1* gene sets from two AML series

**Figure 1:**
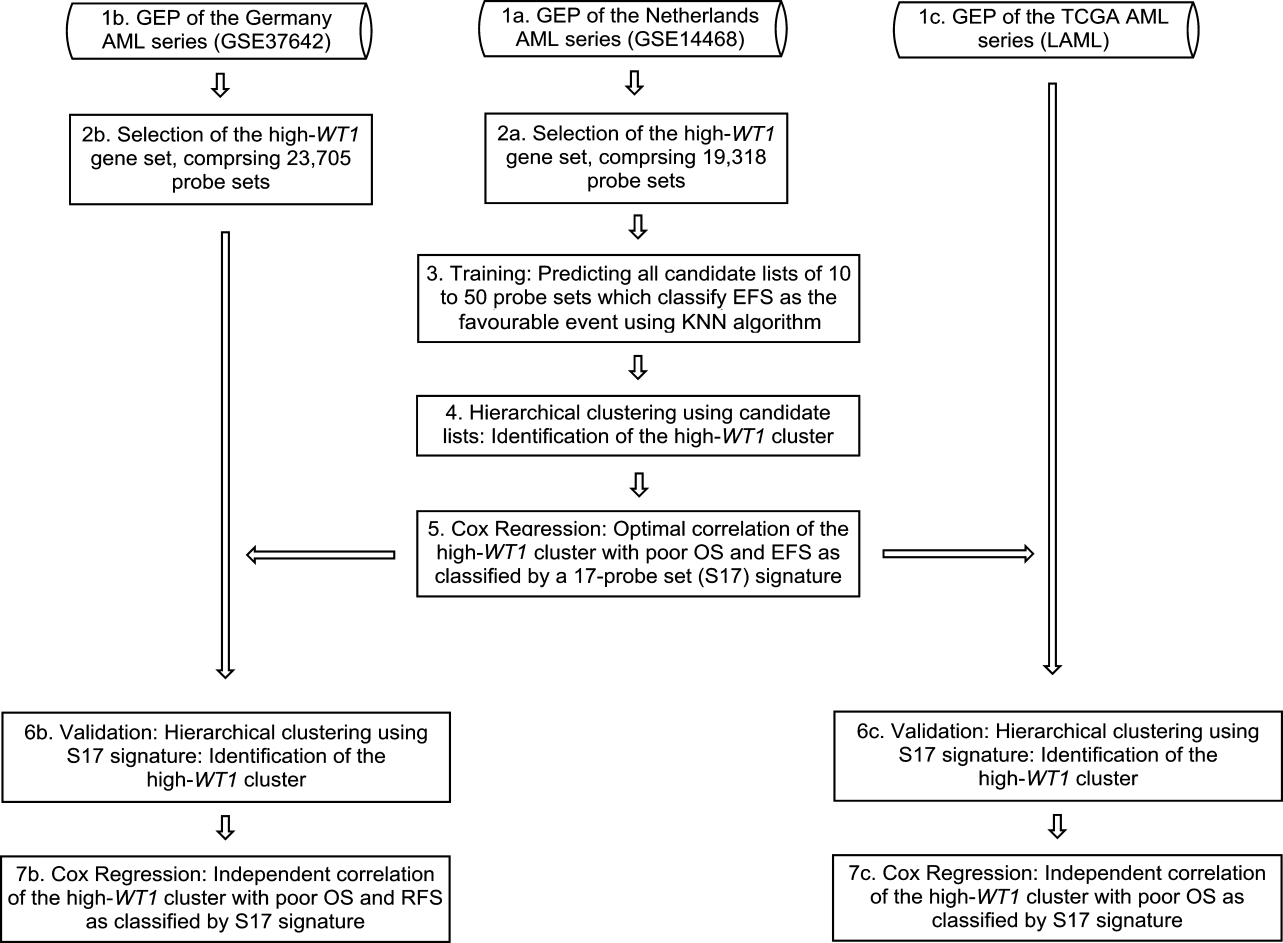
The workflow used to identify and validate the prognostic gene signature associated with high levels of *WT1* expression. AML, acute myeloid leukaemia; GEP, gene expression profiling; OS, overall survival; EFS, event‐free survival; RFS, relapse‐free survival; TCGA, The Cancer Genome Atlas.

Normalized gene expression data were used to obtain differentially expressed genes in high *WT1* expressing samples. Comparison of the high *WT1* (Q4) and low *WT1* (Q1) samples in the Netherlands series (Fig 1) identified 19 318 probe‐sets as differentially expressed (Table SI), including *WT1* expression level showing 10·3 fold difference. Likewise, 23 705 probe‐sets were found to be differentially expressed in high *WT1* samples in the Germany series (Table SII), including *WT1* expression level, which showed 16·4‐fold difference. These probe‐sets represented genes that were either positively or negatively correlated with *WT1* expression levels. About 62% of the probe‐sets in the Netherlands high *WT1* gene set were also found in the Germany high *WT1* set, with 96·7% of them differing in the same direction. Linear regression analysis showed a high degree of correlation of the fold differences between the two high *WT1* sets (Fig S1). At the gene level, 57% similarity was observed between the top 100 genes from the two high *WT1* gene sets (Tables SI and SII).

### A 17‐probe set signature was found to be the optimal predictor of prognosis in the Netherlands AML series

**Figure 2:**
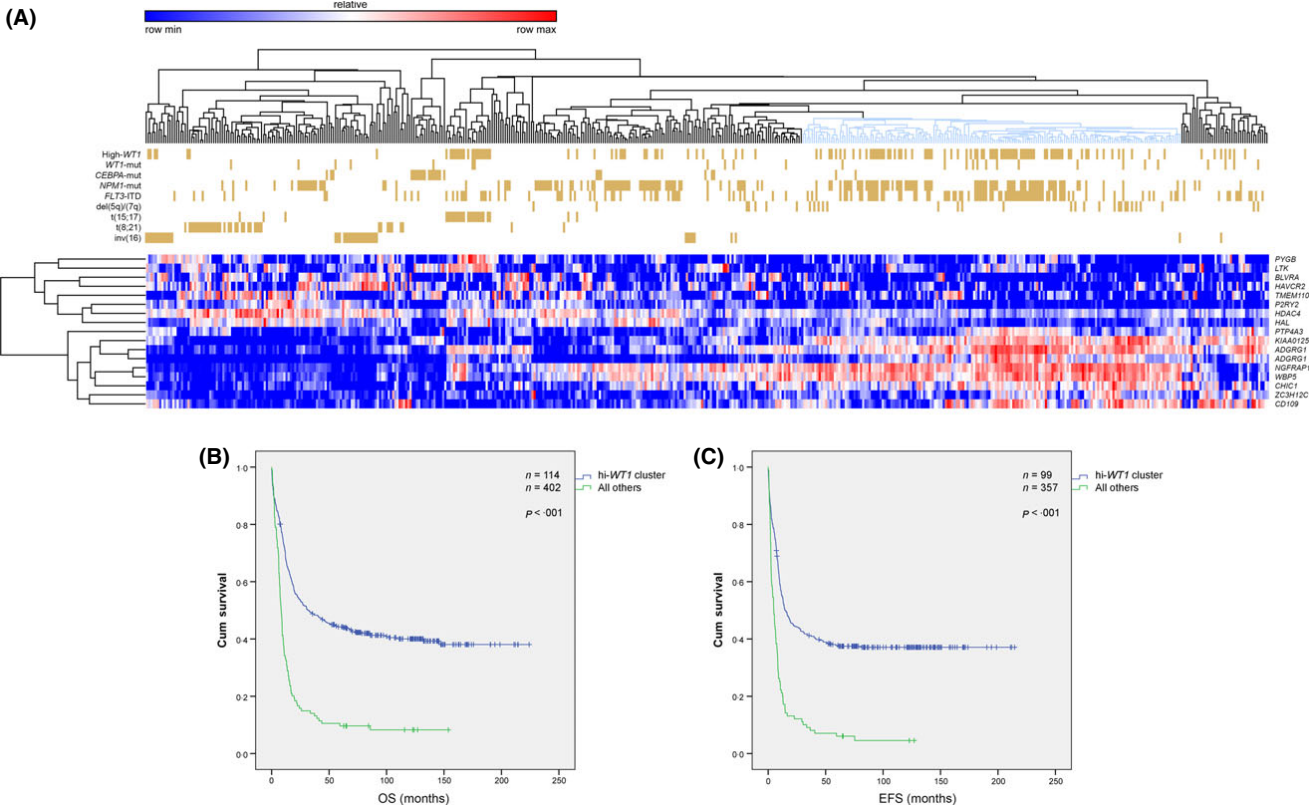
(A) Supervised clustering of the Netherlands acute myeloid leukaemia (AML) series using the S17 signature. The marked cluster of the high *WT1* comprising of 34% of the patients with AML showed distinct gene expression profiling as compared to the remaining clusters. This cluster was found to be positively associated with del(5q)/(7q), *FLT3*‐internal tandem duplication (ITD) and *WT1* mutational status, whereas it was negatively associated with inv(16), t(15;17), t(8;21), and *CEBPA* double mutation, with the latter markers creating distinct clusters. (B, C) Kaplan–Meier analysis of the survival in the training Netherland series clustered by S17 signature. Log‐Rank (Mantel–Cox) *P*‐values included 4·4 × 10^−18^ and 2·1 × 10^−16^ for OS (B) and EFS (C), respectively. OS, overall survival; EFS, event‐free survival;

The workflow that was used for prediction training and validation is illustrated in Fig 1. Briefly, The Netherlands GEP limited to the high *WT1* set was used as a classifier by means of KNNXValidation and EFS as the favourable event. GEP of the AML samples were clustered using lists of 10 to 50 probe‐sets as predicted by KNNXValidation. Classification of the Netherlands AML series using each predicted list identified a cluster of patients with distinct GEP that was associated with high *WT1* expression levels (Q4). This high *WT1* cluster was found to be associated with adverse prognosis, with a list of 17 probe‐sets (herein called S17 signature) as the optimal predictor of the long‐term prognosis in terms of both significance level and hazard ratio (HR) (Fig S2). The S17 signature (Table SIII) classified a distinct cluster of patients (Fig 2A) associated with high *WT1* status [Odds ratio (OR) =3·0, *P *=* *1·6 × 10^−6^] as well as poor prognosis, with 5‐year OS of 9·6% vs. 44·1% {HR = 2·72 [95% confidence interval (CI): 2·15–3·44]}, and 5‐year EFS of 6·1% vs. 37·7% (HR = 2·69 [95% CI: 2·10–3·44]) (Fig 2B–C, Table SIV). The median OS and EFS for this cluster of patients were 8·3 (95% CI: 6·7–9·9) and 4·9 (95% CI: 3·2–6·5) months respectively, compared to 32·3 (95% CI: 16·81–47·8) and 14·5 (95% CI: 9·4–19·5) months for others. Although the high *WT1* cluster was also found to be correlated to other known risk factors including del(5q)/(7q), *FLT3*‐internal tandem duplication (ITD), *WT1* status (positively), and inv(16), t(15;17), t(8;21), *FLT3*‐tyrosine kinase domain (TKD) and *CEBPA* (double mutation) status (negatively) (Fig 2A), its prognostic impact remained highly significant after adjustment for baseline characteristics and known prognostic factors (Table 1).

**Table 1:** Multivariate analysis of the OS and EFS in the Netherlands acute myeloid leukaemia series using Cox Regression analysis of those variables which were significant at the level of *P* < 0.1 in univariate Cox Regression

Variable	OS				EFS			
	*P*‐value	HR	95% CI for HR		*P*‐value	HR	95% CI for HR	
			Lower	Upper			Lower	Upper
S17 signature	<0·001	1·852	1·387	2·473	<0·001	1·934	1·465	2·555
Age	0·026	1·121	1·013	1·239	NS	‐	‐	‐
Complex karyotype	0·001	2·198	1·397	3·458	0·001	2·182	1·354	3·518
del(5q)/del(7q)	0·017	1·76	1·109	2·795	0·001	2·027	1·322	3·109
inv(16)	0·027	0·537	0·31	0·931	0·036	0·569	0·336	0·964
t(8;21)	0·010	0·465	0·259	0·833	0·004	0·444	0·254	0·775
*FLT3*‐ITD	0·004	1·526	1·142	2·037	0·003	1·548	1·161	2·062
*NPM1*‐mutated	0·001	0·615	0·456	0·83	0·001	0·603	0·452	0·804
*CEBPA*‐double‐mutated	0·008	0·397	0·201	0·785	0·008	0·4	0·203	0·789

OS, overall survival; EFS, event‐free survival; HR, Hazard ratio.

### The S17 signature was an independent prognostic factor in the Germany AML series

**Figure 3:**
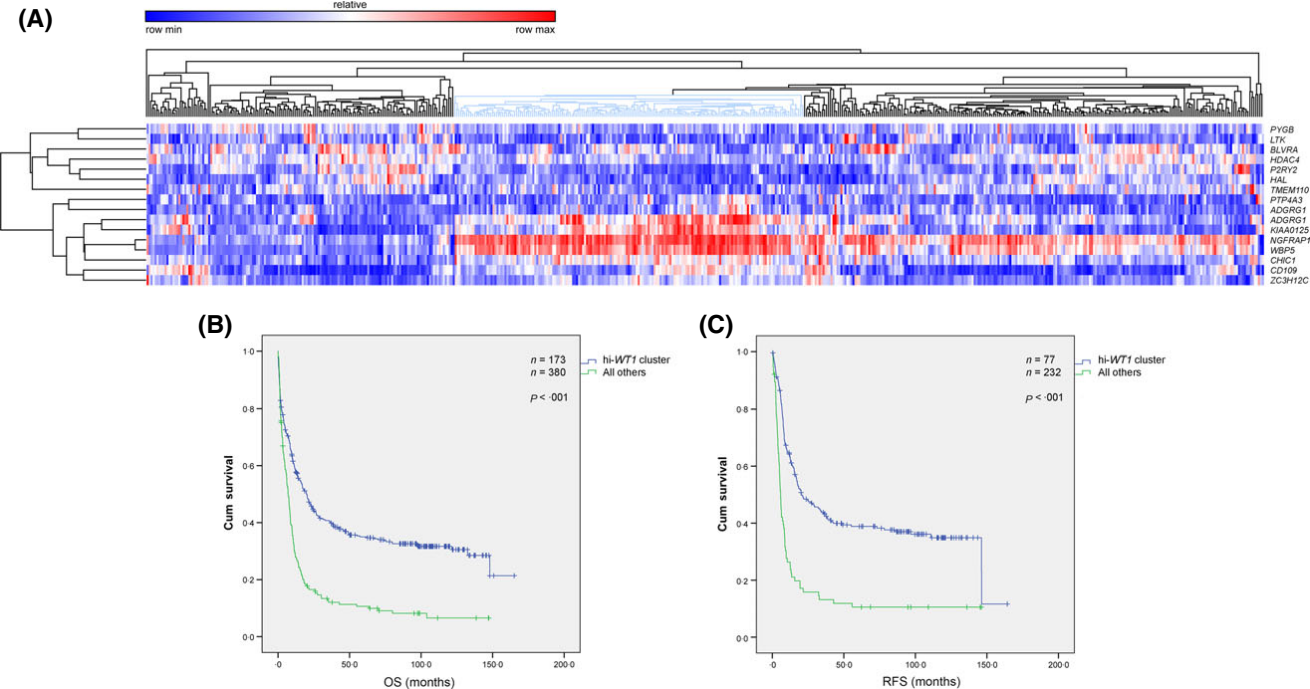
(A) Supervised clustering of the Germany acute myeloid leukaemia series using the S17 signature. The marked cluster of the high *WT1* patients, comprising 31% of the cases, showed distinct gene expression profiling as compared to the remaining clusters. (B, C) Kaplan–Meier analysis of the survival in the Germany validation series clustered by S17 signature. Log‐Rank (Mantel–Cox) *P*‐values included 2·2 × 10^−13^ and 5·4 × 10^−12^ for OS (B) and RFS (C), respectively. OS, overall survival; RFS, relapse‐free survival;

In order to validate the prognostic value of the S17 signature, it was tested against the Germany series. Supervised clustering of the Germany series using the gene signature predicted a cluster of patients with a distinct GEP similar to the Netherlands series (Fig 3A) and predicting both adverse OS and RFS (Fig 3B–C, Table SV). The median OS was 7·6 (95% CI: 5·9–9·3) months for the cluster of patients with high *WT1* as compared to 13·9 (95% CI: 11·0–16·8) months for other cases. Similarly, the median RFS was 5·3 (95% CI: 4·2–6·4) months for high *WT1* cases as compared to 17·3 (95% CI: 13·4–21·2) months for the remaining patients. This prognostic impact of the S17 signature remained statistically significant after adjustment for baseline characteristics and ELN genetic risk groups (Table 2).

**Table 2:** Multivariate analysis of the OS and RFS in the Germany AML series using Cox Regression analysis of the ELN genetic groups and those variables which were significant at the level of *P *< 0·1 in univariate Cox Regression

Variable	OS				RFS			
	*P*‐value	HR	95% CI for HR		*P*‐value	HR	95% CI for HR	
			Lower	Upper			Lower	Upper
S17 signature	<0·001	1·485	1·186	1·860	<0·001	1·834	1·326	2·536
Age	<0·001	1·291	1·197	1·391	<0·001	1·198	1·081	1·327
ELN2^a^	<0·001	2·395	1·743	3·291	<0·001	3·147	2·124	4·663
ELN3^a^	<0·001	2·395	1·732	3·312	<0·001	2·153	1·405	3·299
ELN4^a^	<0·001	3·306	2·376	4·599	<0·001	6·401	4·027	10·175

OS, overall survival; RFS, relapse‐free survival; HR, Hazard ratio.

a The ELN2, ELN3, and ELN4 indicate the corresponding genetic ELN groups compared to the ELN1 group, respectively.

### The S17 signature predicted adverse survival in the TCGA AML series

**Figure 4:**
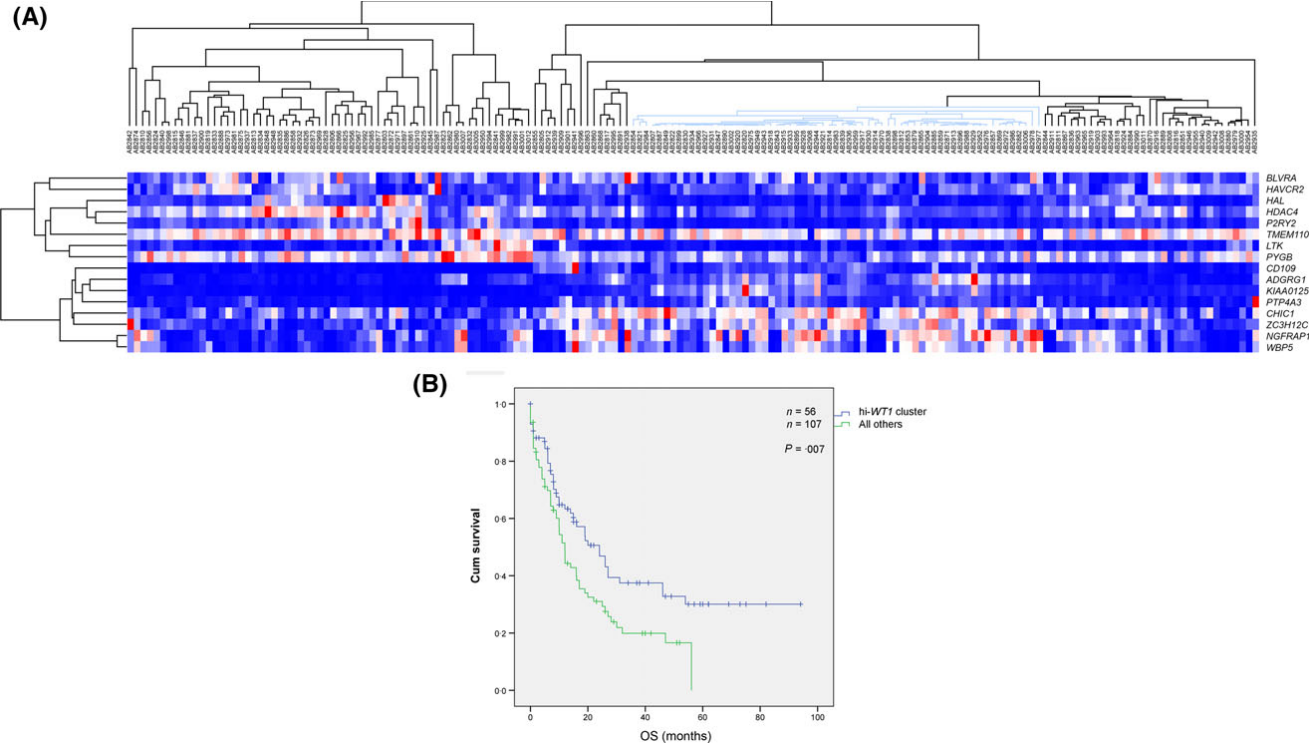
(A) Supervised clustering of the The Cancer Genome Atlas (TCGA) acute myeloid leukaemia (AML) series using the S17 signature. The marked cluster of the high *WT1* patients, comprising 36% of the cases, showed distinct gene expression profiling as compared to the remaining clusters. (B) Kaplan–Meier analysis of the survival in the TCGA validation series clustered by S17 signature. Log‐Rank (Mantel–Cox) *P*‐value was 6·9 × 10^−3^ for OS.

We further tested the prognostic value of the high *WT1* signature in the TCGA series, which showed a cluster of patients with a similar distinct GEP (Fig 4A), predicting adverse OS (Fig 4B). The median OS was 12·0 (95% CI: 10·1–13·9) months for the cluster of patients with high *WT1* as compared to 19·1 (95% CI: 11·2–27·0) months for others. No correlation was found between the high *WT1* cluster and gene mutations of prognostic significance, including *FLT3*‐ITD*, NPM1,* or *IDH* mutations. Immunophenotyping analysis of the AML cells identified a positive correlation of the high *WT1* cluster with CD34^+^ (OR = 4·5, *P *=* *0·001) and CD7^+^ (OR = 14·4, *P *=* *0·03) cells.

### An expression score based on top four genes predicted adverse outcome in three AML series

**Figure 5:**
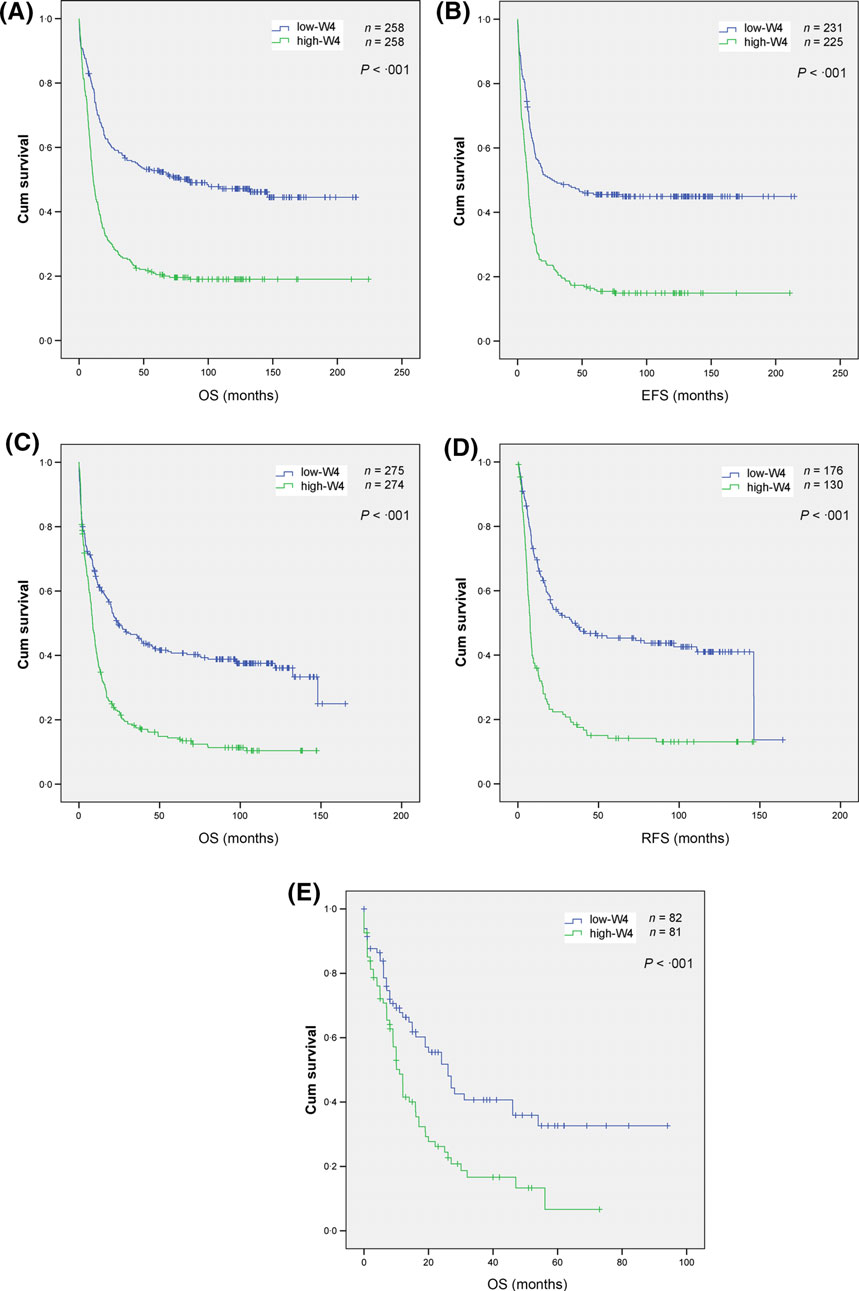
Kaplan–Meier analysis of the survival in three acute myeloid leukaemia (AML) series as stratified using W4 gene expression score. (A, B) Survival analysis in the Netherlands AML series. (C, D) Survival analysis in the Germany series. (E) Survival analysis in The Cancer Genome Atlas series. OS, overall survival; EFS, event‐free survival; RFS, relapse‐free survival.

ROC curve analysis ranked 17 probe‐sets based on their correlation to the EFS in the Netherlands series (Table SVI). *CD109* (226545_at) was the most significant gene and the *HAVCR2* (1555628_a_at) the least significant. A gene expression score derived from the cumulative expression levels of the top four genes [*CD109, KIAA0125* (also termed *FAM30A*)*, NGFRAP1* and *ZC3H12C*], herein called W4, demonstrated very high correlation to the EFS in the Netherlands series (Table SVII). The W4 genes were all overexpressed in the poor risk group. As anticipated, the W4 score predicted adverse outcome in the Netherlands series tested by Cox regression analysis (Fig 5A), with a median OS of 10·9 and 85·5 months (*P *=* *2 × 10^−14^) in the high‐ and low‐W4 cases, respectively. Similarly, the median EFS was 8·0 and 26·7 months (*P *=* *4 × 10^−13^) in these groups (Fig 5B), and remained statistically significant after adjustment for baseline characteristics and known prognostic factors (Table SVIII). The W4 expression score was also able to predict adverse outcome in the Germany series. The median OS was 8·5 and 24·5 (*P *=* *2 × 10^−12^) among the high‐ and low‐W4 cases, respectively, and the median RFS was 7·6 and 33·7 months (*P *=* *3 × 10^−11^) in these groups (Fig 5C, D). This prognostic impact remained statistically significant after adjustment for baseline characteristics and ELN genetic groups (Table SIX). The positive predictive value (PPV) and negative predictive value (NPV) of the marker for prediction of adverse OS were 85·8% and 39·3%, respectively. They were found to be 84·6% and 43·8%, respectively, for prediction of adverse RFS. Finally, the W4 score was found to be predictive of adverse OS in the TCGA series. The median OS was found to be 11·0 and 26·0 months (*P *=* *7 × 10^−4^) among high‐ and low‐W4 cases, respectively (Fig 5E). The PPV and NPV of the marker for prediction of adverse OS were 75·3% and 46·3%, respectively.

### Pathway analysis

MetaCore pathway analysis of the Netherlands high *WT1* set of >2‐fold difference identified Antigen Presentation by major histocompatibility complex (MHC) Class II as the top relevant pathway (Fig S3). Most of the pathway genes were found to show low expression, indicating the downregulation of the whole pathway. Likewise, Antigen Presentation by MHC‐II was found as the most biologically relevant pathway in the Germany high *WT1* set of >2‐fold difference (FDR = 8·5 × 10^−3^). MetaCore analysis of the S17 plus WT1 identified a single dense network, indicating their frequent interactions (Fig S4). Three canonical pathways were identified, including those terminating on NFKB1, CREB1, and MEF2 transcription factors. The upregulated HDAC4 seemed to be in a hub position for the network.

## Discussion

Several studies have attempted to risk‐stratify AML patients using gene signatures obtained from GEP, though most of these signatures were correlated with known risk factors and hence of no further clinical benefit, including those associated with mutations in *NPM1* (Verhaak *et al*, 2005), double mutant *CEBPA* (Wouters *et al*, 2009) and *FLT3‐*ITD (Neben *et al*, 2005). Metzeler *et al* (2008) used an unbiased approach to identify all genes whose expression levels were correlated to the OS, which led to a prognostic score based on a 66‐gene signature. More recently, Li *et al* (2013) conducted a similar global study with more extensive training and validation steps and identified a 24‐gene signature that improved ELN risk classification of AML. On the other hand, Becker *et al* (2010) described a signature of 131 genes that was associated with the *WT1* mutation, though without any clinical impact.

We showed here that differentially expressed genes in high *WT1* samples were highly correlated in two AML series in terms of altered genes, expression profiles and implicated pathways, collectively corroborating the biological relevance of the high *WT1* gene signature. Hence, we inferred a 16‐gene signature using differentially expressed genes in high *WT1* samples that was able to predict AML outcome in two independent AML series. Although the S17 signature was also associated with other known risk factors including some cytogenetic aberrations, *FLT3*‐ITD and *NPM1* status, as anticipated from a marker which is overexpressed in the vast majority of AML samples (Ostergaard *et al*, 2004), it was also predictive of clinical outcome independently of the currently accepted ELN genetic groups (Dohner *et al*, 2010). This might reflect the prognostic value of *WT1* expression level in the vast majority of AML patients regardless of their underlying genetic risk factors (Bergmann *et al*, 1997; Trka *et al*, 2002).

*WT1* mutation has been controversially reported to be associated with adverse prognosis in AML (Virappane *et al*, 2008; Gaidzik *et al*, 2009). We did not observe any prognostic impact of *WT1* mutation in the Netherlands series. However, given the association of the *WT1* mutation with the S17 signature, one cannot rule out the possibility of its indirect impact through *WT1* expression level. Given the growing list of gene mutations of prognostic implication in AML, including recently identified mutations in *DNMT3A, TET2,* and *ASXL1*, a good surrogate marker can be a more global one, such as the S17 signature, which is correlated to several markers, in addition to its independent impact. We also identified a CD34^+^‐CD7^+^ phenotype for high *WT1* cluster of AML cells, which has already been found to be associated with poor risk in AML (Del Poeta *et al*, 1995) and possibly involved in clonal evolution of CML (Kosugi *et al*, 2005). However, the implication of this finding needs further investigation.

Antigen Presentation by MHC Class II was found to be the most relevant biological pathway in our study, which is in line with findings of Wilson *et al* (2006), who demonstrated that a cluster of AML patients with high *WT1* expression also showed low expression of MHC‐II genes. The fact that targetable HDAC4 has been found to be a master regulator of the S17 network might be of potential therapeutic relevance [reviewed in (Tan *et al*, 2010)].

Finally, the S17 signature consistently predicted long term outcome in different clinical settings, including age groups, karyotype status and a wide variety of treatment regimens. The S17 signature also demonstrated a similar prognostic value in the much smaller series of TCGA‐AML samples assessed using a different GEP platform, i.e. RNA‐seq, demonstrating robust cross‐platform predictive value. This was particularly promising, given that the RNA‐seq gene expression data show broad dynamic range (Zhao *et al*, 2014) and a very high correlation to qRT‐PCR results (Rapaport *et al*, 2013). We hence attempted to derive a gene expression score using the most significant genes amongst those included in the S17 signature. This led to the 4‐gene W4 score, which faithfully predicted the adverse outcome in the TCGA series tested by RNA‐seq, as well as the other two AML series examined by microarray analyses. Therefore, the W4 score can be easily used in a short AML series, or in its normalized form, as a surrogate marker for the prognostication of AML patients on a day‐to‐day basis. Among the W4 genes, *CD109,* which is also found in the gene signature reported by Metzeler *et al* (2008), is known to be expressed on a subset of haematopoietic stem and progenitor cells (Lin *et al*, 2002) as well as activated platelets and T‐lymphoblasts (Sutherland *et al*, 1991), corroborating our immunophenotyping findings. *CD109* overexpression has also been observed in many human cancers (Hashimoto *et al*, 2004). The other overexpressed gene, *NGFRAP1,* belongs to the neurotrophin signalling pathway, of which many genes, including *NTRK1, NTRK2* and *NTRK3,* are known to be implicated in leukaemogenesis (Eguchi *et al*, 1999; Meyer *et al*, 2007; Li *et al*, 2009). To our knowledge, the W4 risk score is the simplest gene expression score predicting long‐term AML prognosis (Gentles *et al*, 2010; Bou Samra *et al*, 2012; Marcucci *et al*, 2014).

In conclusion, we identified a short gene signature associated with high *WT1* expression and demonstrated its adverse and independent prognostic impact in adult AML patients. A 4‐gene expression score was next derived, which similarly predicted AML prognosis in the three series examined. Our study proposes a novel way to incorporate a candidate prognostic factor, i.e. *WT1* expression level, into the current models of AML risk stratification and provides new clues to the molecular mechanisms underlying *WT1* regulation. These promising results will have to be validated in further trials.

## Author contributions

AN performed the research and wrote the manuscript; AN and DB designed the research study; AN and TH analysed the data; TH, YR, MCS, TB, WH, SKB, PJMV, and DB contributed essential data and reviewed the manuscript.

## Supporting information


**Fig S1.** Differential gene expression in shared probe sets among Netherlands and Germany high‐*WT1* gene sets. The differences in expression levels were significantly correlated among two gene sets (*r* ^2^ = 0.81, *P *< 10^−18^).
**Fig S2.** Univariate survival analysis of the supervised clustering by candidate lists of probe sets in order to obtain the optimal survival predictor.
**Fig S3.** Antigen Presentation by MHC Class II as the most significant MetaCore pathway deregulated in Netherlands 2‐fold high‐*WT1* set (826 probe sets). Those genes with an adjacent color bar showed differed expression in high‐*WT1* state, with the height of the bar as the relative decrease (blue) or increase (red) in expression level of the gene. Most key genes showed decreased expression, and hence the entire pathway seems to be downregulated in high‐*WT1*.
**Fig S4.** MetaCore Shortest Network as defined by the network formed among the S17 gene products and WT1, with not more than one connection.
**Table SI.** Top 100 genes differentially expressed between the highest (Q4) and lowest (Q1) quartiles of *WT1* expression level in the Netherlands series, or Netherlands high‐*WT1* set.
**Table SII.** Top 100 genes diffentially expressed between the highest (Q4) and lowest (Q1) quartiles of *WT1* expression level in the Germany series, or Germany high‐*WT1* set.
**Table SIII.** The S17 signature (consisting of 16 unique gene transcripts) which optimally classified the high‐WT1 cluster associated with poor prognosis in Netherlands high‐*WT1* state.
**Table SIV.** Univariate analysis of the OS and EFS in the Netherlands AML series using Cox Regression analysis of the basic covariates and cytogenetic and molecular aberrations.
**Table SV.** Univariate analysis of the OS and RFS in Germany AML series using Cox Regression analysis of all potentially implicated variables.
**Table SVI.** Correlation of the individual S17 probe sets to the EFS in the Netherlands series using ROC curve analysis. Those probe‐sets with AUCs (area under the curve) significantly above 0.5 correlate positively with the EFS, while those with AUCs less than 0.5 correlate negatively.
**Table SVII.** Correlation of the gene scores obtained from cumulative gene expression of the *n* most significant probe‐sets of the S17 to the EFS in the Netherlands series using ROC curve analysis. Both the AUC (area under the curve) and the *P*‐value did change little after W4.
**Table SVIII.** Multivariate analysis of the OS and EFS in the Netherlands AML series using Cox Regression analysis of those variables which were significant at the level of *P* < 0.1 in univariate Cox Regression.
**Table SXI.** Multivariate analysis of the OS and RFS in Germany AML series using Cox Regression analysis of the ELN genetic groups and those variables which were significant at the level of *P* < 0.1 in univariate Cox Regression.
