# Supplementary material for: A 4‐gene expression score associated with high levels of Wilms Tumor‐1 (WT1) expression is an adverse prognostic factor in acute myeloid leukaemia
Source: Br J Haematol. 2015 Nov 24;172(3):401–11. doi: 10.1111/bjh.13836 (PMC4833185; doi:10.1111/bjh.13836)

**A 4-Gene Expression Score Associated with High Levels of *Wilms Tumor-1* (*WT1*) Expression Is an Adverse Prognostic Factor in Acute Myeloid Leukemia**

Table S1. Top 100 genes differentially expressed between the highest (Q4) and lowest (Q1) quartiles of *WT1* expression level in the Netherlands series, or Netherlands high-*WT1* set. For those genes represented by more than one probe set, just one is indicated here.

| **Probeset** | **Gene symbol** | **Gene Name** | **Netherlands fold dif.** | **FDR** |
| --- | --- | --- | --- | --- |
| **Top 50 upregulated** |  |  |  |  |
| 206067_s_at | *WT1* | Wilms tumor 1 | 10.34 | 2.3E-170 |
| 205624_at | *CPA3* | carboxypeptidase A3 (mast cell) | 6.40 | 2.1E-23 |
| 201069_at | *MMP2* | matrix metallopeptidase 2 (gelatinase A, 72kDa gelatinase, 72kDa type IV collagenase) | 5.71 | 7.1E-29 |
| 201427_s_at | *SEPP1* | selenoprotein P, plasma, 1 | 5.19 | 4.8E-16 |
| 238021_s_at | *CRNDE* | colorectal neoplasia differentially expressed (non-protein coding) | 5.08 | 2.8E-26 |
| 202718_at | *IGFBP2* | insulin-like growth factor binding protein 2, 36kDa | 4.94 | 3.3E-23 |
| 229638_at | *IRX3* | iroquois homeobox 3 | 4.84 | 6.8E-21 |
| 226134_s_at | *MSI2* | musashi RNA-binding protein 2 | 4.81 | 1.9E-31 |
| 213395_at | *MLC1* | megalencephalic leukoencephalopathy with subcortical cysts 1 | 4.72 | 2.5E-49 |
| 213110_s_at | *COL4A5* | collagen, type IV, alpha 5 | 4.69 | 5.0E-23 |
| 201596_x_at | *KRT18* | keratin 18 | 4.60 | 8.1E-27 |
| 205899_at | *CCNA1* | cyclin A1 | 4.53 | 3.5E-21 |
| 236738_at | *LOC401097* | chromosome 3 open reading frame 80 | 4.36 | 1.6E-24 |
| 231982_at | *C19orf77* | chromosome 19 open reading frame 77 | 4.34 | 2.8E-22 |
| 206310_at | *SPINK2* | serine peptidase inhibitor, Kazal type 2 (acrosin-trypsin inhibitor) | 4.30 | 4.2E-15 |
| 1552908_at | *C1orf150* | germinal center-associated, signaling and motility-like | 4.02 | 7.6E-35 |
| 205609_at | *ANGPT1* | angiopoietin 1 | 3.90 | 3.3E-16 |
| 206478_at | *KIAA0125* | KIAA0125 | 3.81 | 1.5E-18 |
| 209710_at | *GATA2* | GATA binding protein 2 | 3.73 | 1.7E-30 |
| 228885_at | *MAMDC2* | MAM domain containing 2 | 3.71 | 2.6E-18 |
| 212775_at | *OBSL1* | obscurin-like 1 | 3.66 | 1.6E-31 |
| 209757_s_at | *MYCN* | v-myc myelocytomatosis viral related oncogene, neuroblastoma derived (avian) | 3.59 | 2.6E-16 |
| 206660_at | *IGLL1* | immunoglobulin lambda-like polypeptide 1 | 3.55 | 1.9E-14 |
| 212070_at | *ADGRG1* | adhesion G protein-coupled receptor G1 | 3.54 | 2.4E-18 |
| 201564_s_at | *FSCN1* | fascin homolog 1, actin-bundling protein (Strongylocentrotus purpuratus) | 3.54 | 1.4E-31 |
| 228988_at | *ZNF711* | zinc finger protein 711 | 3.50 | 1.0E-17 |
| 227556_at | *NME7* | NME/NM23 family member 7 | 3.49 | 5.0E-27 |
| 220377_at | *KIAA0125* | FAM30A | 3.49 | 1.0E-17 |
| 201242_s_at | *ATP1B1* | ATPase, Na+/K+ transporting, beta 1 polypeptide | 3.47 | 1.7E-24 |
| 226043_at | *GPSM1* | G-protein signaling modulator 1 | 3.43 | 1.9E-27 |
| 227860_at | *CPXM1* | carboxypeptidase X (M14 family), member 1 | 3.41 | 1.2E-19 |
| 1552623_at | *HSH2D* | hematopoietic SH2 domain containing | 3.39 | 7.9E-29 |
| 202746_at | *ITM2A* | integral membrane protein 2A | 3.36 | 1.3E-14 |
| 214450_at | *CTSW* | cathepsin W | 3.36 | 5.4E-15 |
| 213668_s_at | *SOX4* | SRY (sex determining region Y)-box 4 | 3.35 | 1.4E-17 |
| 217963_s_at | *NGFRAP1* | nerve growth factor receptor (TNFRSF16) associated protein 1 | 3.34 | 1.0E-11 |
| 216268_s_at | *JAG1* | jagged 1 | 3.33 | 2.8E-21 |
| 209409_at | *GRB10* | growth factor receptor-bound protein 10 | 3.18 | 7.1E-22 |
| 235142_at | *ZBTB8A* | zinc finger and BTB domain containing 8A | 3.17 | 1.3E-19 |
| 205780_at | *BIK* | BCL2-interacting killer (apoptosis-inducing) | 3.15 | 3.8E-27 |
| 238784_at | *DPY19L2* | dpy-19-like 2 (C. elegans) | 3.14 | 1.4E-22 |
| 204044_at | *QPRT* | quinolinate phosphoribosyltransferase | 3.09 | 3.6E-39 |
| 221942_s_at | *GUCY1A3* | guanylate cyclase 1, soluble, alpha 3 | 3.05 | 8.0E-14 |
| 227522_at | *CMBL* | carboxymethylenebutenolidase homolog (Pseudomonas) | 3.03 | 4.7E-24 |
| 205801_s_at | *RASGRP3* | RAS guanyl releasing protein 3 (calcium and DAG-regulated) | 3.03 | 1.4E-18 |
| 204011_at | *SPRY2* | sprouty homolog 2 (Drosophila) | 2.99 | 7.2E-22 |
| 213541_s_at | *ERG* | v-ets erythroblastosis virus E26 oncogene homolog (avian) | 2.99 | 2.8E-20 |
| 205349_at | *GNA15* | guanine nucleotide binding protein (G protein), alpha 15 (Gq class) | 2.97 | 3.1E-39 |
| 228654_at | *SPIN4* | spindlin family, member 4 | 2.97 | 1.6E-33 |
| 214228_x_at | *TNFRSF4* | tumor necrosis factor receptor superfamily, member 4 | 2.97 | 4.1E-22 |
|  |  |  |  |  |
| **Top 50 downregulated** |  |  |  |  |
| 224356_x_at | *MS4A6A* | membrane-spanning 4-domains, subfamily A, member 6A | 0.13 | 6.6E-33 |
| 215646_s_at | *VCAN* | versican | 0.13 | 5.2E-17 |
| 226818_at | *MPEG1* | macrophage expressed 1 | 0.14 | 2.1E-22 |
| 203535_at | *S100A9* | S100 calcium binding protein A9 | 0.15 | 2.1E-16 |
| 201743_at | *CD14* | CD14 molecule | 0.16 | 1.4E-16 |
| 212192_at | *KCTD12* | potassium channel tetramerisation domain containing 12 | 0.16 | 1.7E-23 |
| 209949_at | *NCF2* | neutrophil cytosolic factor 2 | 0.16 | 2.5E-24 |
| 213566_at | *RNASE6* | ribonuclease, RNase A family, k6 | 0.18 | 6.2E-22 |
| 218454_at | *PLBD1* | phospholipase B domain containing 1 | 0.18 | 4.7E-17 |
| 227265_at | *FGL2* | fibrinogen-like 2 | 0.18 | 3.5E-20 |
| 202917_s_at | *S100A8* | S100 calcium binding protein A8 | 0.18 | 3.4E-15 |
| 205789_at | *CD1D* | CD1d molecule | 0.19 | 3.0E-27 |
| 205863_at | *S100A12* | S100 calcium binding protein A12 | 0.19 | 1.2E-16 |
| 212671_s_at | *HLA-DQA1/ HLA-DQA2 /LOC100509457* | major histocompatibility complex, class II, DQ alpha 1 /// major histocompatibility complex, class II, DQ alpha 2 /// HLA class II histocompatibility antigen, DQ alpha 1 chain-like | 0.19 | 2.6E-25 |
| 212998_x_at | *HLA-DQB1/ LOC100294318* | major histocompatibility complex, class II, DQ beta 1 /// HLA class II histocompatibility antigen, DQ beta 1 chain-like | 0.19 | 1.4E-22 |
| 205936_s_at | *HK3* | hexokinase 3 (white cell) | 0.20 | 1.2E-19 |
| 229560_at | *TLR8* | toll-like receptor 8 | 0.20 | 1.4E-21 |
| 205237_at | *FCN1* | ficolin (collagen/fibrinogen domain containing) 1 | 0.20 | 8.7E-16 |
| 201422_at | *IFI30 / PIK3R2* | interferon, gamma-inducible protein 30 /// phosphoinositide-3-kinase, regulatory subunit 2 (beta) | 0.20 | 7.0E-17 |
| 218559_s_at | *MAFB* | v-maf musculoaponeurotic fibrosarcoma oncogene homolog B (avian) | 0.21 | 1.5E-14 |
| 217762_s_at | *RAB31* | RAB31, member RAS oncogene family | 0.21 | 1.9E-25 |
| 211991_s_at | *HLA-DPA1* | major histocompatibility complex, class II, DP alpha 1 | 0.22 | 5.2E-21 |
| 206978_at | *CCR2* | chemokine (C-C motif) receptor 2 | 0.22 | 9.2E-25 |
| 201669_s_at | *MARCKS* | myristoylated alanine-rich protein kinase C substrate | 0.22 | 2.0E-18 |
| 235964_x_at | *SAMHD1* | SAM domain and HD domain 1 | 0.22 | 2.4E-23 |
| 200782_at | *ANXA5* | annexin A5 | 0.22 | 1.9E-18 |
| 1555745_a_at | *LYZ* | lysozyme | 0.23 | 1.2E-15 |
| 214084_x_at | *NCF1C* | neutrophil cytosolic factor 1C pseudogene | 0.23 | 6.3E-18 |
| 205898_at | *CX3CR1* | chemokine (C-X3-C motif) receptor 1 | 0.23 | 4.1E-15 |
| 210982_s_at | *HLA-DRA* | major histocompatibility complex, class II, DR alpha | 0.23 | 5.5E-19 |
| 223204_at | *FAM198B* | family with sequence similarity 198, member B | 0.24 | 1.3E-16 |
| 203645_s_at | *CD163* | CD163 molecule | 0.24 | 1.8E-17 |
| 220088_at | *C5AR1* | complement component 5a receptor 1 | 0.24 | 1.3E-14 |
| 1559502_s_at | *LRRC25* | leucine rich repeat containing 25 | 0.24 | 1.6E-24 |
| 204961_s_at | *NCF1 / NCF1B / NCF1C* | neutrophil cytosolic factor 1 /// neutrophil cytosolic factor 1B pseudogene /// neutrophil cytosolic factor 1C pseudogene | 0.24 | 5.2E-19 |
| 223343_at | *MS4A7* | membrane-spanning 4-domains, subfamily A, member 7 | 0.24 | 1.7E-20 |
| 220532_s_at | *TMEM176B* | transmembrane protein 176B | 0.25 | 4.5E-16 |
| 204057_at | *IRF8* | interferon regulatory factor 8 | 0.25 | 2.4E-27 |
| 207857_at | *LILRA2* | leukocyte immunoglobulin-like receptor, subfamily A (with TM domain), member 2 | 0.25 | 5.9E-30 |
| 208438_s_at | *FGR* | Gardner-Rasheed feline sarcoma viral (v-fgr) oncogene homolog | 0.25 | 8.0E-21 |
| 204588_s_at | *SLC7A7* | solute carrier family 7 (amino acid transporter light chain, y+L system), member 7 | 0.25 | 2.8E-19 |
| 202833_s_at | *SERPINA1* | serpin peptidase inhibitor, clade A (alpha-1 antiproteinase, antitrypsin), member 1 | 0.26 | 5.4E-10 |
| 1555756_a_at | *CLEC7A* | C-type lectin domain family 7, member A | 0.26 | 3.5E-14 |
| 207697_x_at | *LILRB2* | leukocyte immunoglobulin-like receptor, subfamily B (with TM and ITIM domains), member 2 | 0.26 | 3.0E-18 |
| 207104_x_at | *LILRB1* | leukocyte immunoglobulin-like receptor, subfamily B (with TM and ITIM domains), member 1 | 0.26 | 1.9E-23 |
| 219607_s_at | *MS4A4A* | membrane-spanning 4-domains, subfamily A, member 4A | 0.26 | 8.5E-23 |
| 208594_x_at | *LILRA6* | leukocyte immunoglobulin-like receptor, subfamily A (with TM domain), member 6 | 0.26 | 2.8E-25 |
| 205119_s_at | *FPR1* | formyl peptide receptor 1 | 0.27 | 2.3E-11 |
| 212657_s_at | *IL1RN* | interleukin 1 receptor antagonist | 0.27 | 2.1E-16 |
| 222496_s_at | *RBM47* | RNA binding motif protein 47 | 0.27 | 2.4E-23 |

Table S2. Top 100 genes diffentially expressed between the highest (Q4) and lowest (Q1) quartiles of *WT1* expression level in the Germany series, or Germany high-*WT1* set. For those genes represented by more than one probe set, just one is indicated here.

| **Probe set** | **Gene symbol** | **Gene Name** | **Germany fold dif.** | **FDR** |
| --- | --- | --- | --- | --- |
| **Top 50 upregulated** |  |  |  |  |
| 206067_s_at | *WT1* | Wilms tumor 1 | 16.38 | 2.6E-159 |
| 205624_at | *CPA3* | carboxypeptidase A3 (mast cell) | 6.46 | 4.3E-28 |
| 228885_at | *MAMDC2* | MAM domain containing 2 | 5.32 | 1.8E-26 |
| 202746_at | *ITM2A* | integral membrane protein 2A | 4.69 | 1.3E-22 |
| 201069_at | *MMP2* | matrix metallopeptidase 2 (gelatinase A, 72kDa gelatinase, 72kDa type IV collagenase) | 4.23 | 4.6E-31 |
| 201596_x_at | *KRT18* | keratin 18; keratin 18 pseudogene 26; keratin 18 pseudogene 19 | 4.08 | 8.5E-30 |
| 238021_s_at | *CRNDE* | hCG1815491 | 4.07 | 1.5E-20 |
| 201242_s_at | *ATP1B1* | ATPase, Na+/K+ transporting, beta 1 polypeptide | 4.05 | 7.3E-28 |
| 213110_s_at | *COL4A5* | collagen, type IV, alpha 5 | 4.00 | 8.1E-20 |
| 205899_at | *CCNA1* | cyclin A1 | 3.90 | 3.8E-19 |
| 219837_s_at | *CYTL1* | cytokine-like 1 | 3.86 | 2.3E-14 |
| 235142_at | *ZBTB8A* | zinc finger and BTB domain containing 8A | 3.83 | 1.2E-25 |
| 209710_at | *GATA2* | GATA binding protein 2 | 3.80 | 1.6E-36 |
| 225240_s_at | *MSI2* | musashi homolog 2 (Drosophila) | 3.78 | 1.5E-28 |
| 236738_at | *LOC401097* | Similar to LOC166075 | 3.61 | 5.3E-24 |
| 209757_s_at | *MYCN* | v-myc myelocytomatosis viral related oncogene, neuroblastoma derived (avian) | 3.56 | 4.0E-22 |
| 212775_at | *OBSL1* | obscurin-like 1 | 3.18 | 9.7E-27 |
| 211071_s_at | *MLLT11* | myeloid/lymphoid or mixed-lineage leukemia (trithorax homolog, Drosophila); translocated to, 11 | 3.16 | 2.4E-35 |
| 201427_s_at | *SEPP1* | selenoprotein P, plasma, 1 | 3.15 | 5.0E-12 |
| 229530_at | *GUCY1A3* | guanylate cyclase 1, soluble, alpha 3 | 3.10 | 7.7E-17 |
| 228988_at | *ZNF711* | zinc finger protein 711 | 3.06 | 4.1E-09 |
| 231982_at | *C19orf77* | similar to HSPC323 | 3.04 | 1.6E-23 |
| 206478_at | *KIAA0125* | KIAA0125 | 3.03 | 4.3E-14 |
| 242051_at | *CD99* |  | 3.02 | 1.8E-21 |
| 213258_at | *TFPI* | tissue factor pathway inhibitor (lipoprotein-associated coagulation inhibitor) | 3.00 | 2.4E-12 |
| 206310_at | *SPINK2* | serine peptidase inhibitor, Kazal type 2 (acrosin-trypsin inhibitor) | 2.95 | 3.2E-10 |
| 216268_s_at | *JAG1* | jagged 1 (Alagille syndrome) | 2.93 | 5.3E-16 |
| 205349_at | *GNA15* | guanine nucleotide binding protein (G protein), alpha 15 (Gq class) | 2.92 | 4.4E-37 |
| 200986_at | *SERPING1* | serpin peptidase inhibitor, clade G (C1 inhibitor), member 1 | 2.90 | 5.0E-20 |
| 228904_at | *HOXB3* | homeobox B3 | 2.89 | 6.3E-09 |
| 241133_at | *TRBV27* | T cell receptor beta variable 27 | 2.89 | 5.7E-14 |
| 205609_at | *ANGPT1* | angiopoietin 1 | 2.89 | 2.0E-17 |
| 215111_s_at | *TSC22D1* | TSC22 domain family, member 1 | 2.87 | 3.3E-22 |
| 204030_s_at | *SCHIP1* | schwannomin interacting protein 1 | 2.78 | 2.2E-13 |
| 202932_at | *YES1* | v-yes-1 Yamaguchi sarcoma viral oncogene homolog 1 | 2.76 | 1.5E-22 |
| 203787_at | *SSBP2* | single-stranded DNA binding protein 2 | 2.75 | 9.5E-25 |
| 205683_x_at | *TPSAB1* | tryptase alpha/beta 1; tryptase beta 2 | 2.74 | 4.3E-11 |
| 229629_at | *GPR174* |  | 2.73 | 1.4E-25 |
| 206761_at | *CD96* | CD96 molecule | 2.63 | 1.7E-12 |
| 228293_at | *DEPDC7* | DEP domain containing 7 | 2.63 | 2.4E-19 |
| 205051_s_at | *KIT* | similar to Mast/stem cell growth factor receptor precursor (SCFR) (Proto-oncogene tyrosine-protein kinase Kit) (c-kit) (CD117 antigen); v-kit Hardy-Zuckerman 4 feline sarcoma viral oncogene homolog | 2.62 | 9.1E-14 |
| 242028_at | *ZNF709* | zinc finger protein 709 | 2.61 | 1.2E-25 |
| 201416_at | *SOX4* | SRY (sex determining region Y)-box 4 | 2.60 | 3.0E-16 |
| 207134_x_at | *TPSB2* | tryptase alpha/beta 1; tryptase beta 2 | 2.60 | 1.8E-10 |
| 210239_at | *IRX5* | iroquois homeobox 5 | 2.59 | 3.5E-14 |
| 212414_s_at | *GLYR1/ SEPT6* | septin 6 | 2.57 | 9.9E-28 |
| 201564_s_at | *FSCN1* | fascin homolog 1, actin-bundling protein (Strongylocentrotus purpuratus) | 2.56 | 9.2E-31 |
| 213348_at | *CDKN1C* | cyclin-dependent kinase inhibitor 1C (p57, Kip2) | 2.55 | 6.6E-20 |
| 227522_at | *CMBL* | carboxymethylenebutenolidase homolog (Pseudomonas) | 2.53 | 3.9E-22 |
| 226043_at | *GPSM1* | G-protein signaling modulator 1 (AGS3-like, C. elegans) | 2.50 | 4.9E-36 |
|  |  |  |  |  |
| **Top 50 downregulated** |  |  |  |  |
| 205863_at | *S100A12* | S100 calcium binding protein A12 | 0.22 | 5.9E-19 |
| 205789_at | *CD1D* | CD1d molecule | 0.24 | 3.2E-19 |
| 205237_at | *FCN1* | ficolin (collagen/fibrinogen domain containing) 1 | 0.24 | 1.8E-17 |
| 218454_at | *PLBD1* | phospholipase B domain containing 1 | 0.25 | 1.2E-15 |
| 212192_at | *KCTD12* | potassium channel tetramerisation domain containing 12 | 0.26 | 2.0E-14 |
| 204961_s_at | *NCF1 / NCF1B / NCF1C* | neutrophil cytosolic factor 1; neutrophil cytosolic factor 1C pseudogene | 0.27 | 1.9E-22 |
| 201743_at | *CD14* | CD14 molecule | 0.27 | 1.6E-14 |
| 204620_s_at | *VCAN* | versican | 0.27 | 1.5E-10 |
| 217764_s_at | *RAB31* | RAB31, member RAS oncogene family | 0.28 | 1.3E-19 |
| 209949_at | *NCF2* | neutrophil cytosolic factor 2 | 0.28 | 2.9E-25 |
| 208438_s_at | *FGR* | Gardner-Rasheed feline sarcoma viral (v-fgr) oncogene homolog | 0.28 | 2.5E-27 |
| 201669_s_at | *MARCKS* | myristoylated alanine-rich protein kinase C substrate | 0.29 | 7.8E-12 |
| 226818_at | *MPEG1* | macrophage expressed 1 | 0.29 | 1.8E-12 |
| 210146_x_at | *LILRB2* | leukocyte immunoglobulin-like receptor, subfamily B (with TM and ITIM domains), member 2 | 0.30 | 5.6E-18 |
| 220088_at | *C5AR1* | complement component 5a receptor 1 | 0.30 | 8.2E-14 |
| 204614_at | *SERPINB2* | serpin peptidase inhibitor, clade B (ovalbumin), member 2 | 0.31 | 1.9E-11 |
| 222496_s_at | *RBM47* | RNA binding motif protein 47 | 0.31 | 2.3E-20 |
| 203535_at | *S100A9* | S100 calcium binding protein A9 | 0.31 | 9.2E-16 |
| 205627_at | *CDA* | cytidine deaminase | 0.31 | 3.9E-22 |
| 202018_s_at | *LTF* | lactotransferrin | 0.32 | 3.2E-10 |
| 229560_at | *TLR8* | toll-like receptor 8 | 0.32 | 5.7E-16 |
| 220005_at | *P2RY13* | purinergic receptor P2Y, G-protein coupled, 13 | 0.32 | 1.0E-20 |
| 213524_s_at | *G0S2* | G0/G1switch 2 | 0.32 | 1.6E-13 |
| 200782_at | *ANXA5* | annexin A5 | 0.33 | 1.0E-16 |
| 205922_at | *VNN2* | vanin 2 | 0.33 | 1.6E-19 |
| 210873_x_at | *APOBEC3A* | apolipoprotein B mRNA editing enzyme, catalytic polypeptide-like 3A | 0.33 | 6.6E-16 |
| 218559_s_at | *MAFB* | v-maf musculoaponeurotic fibrosarcoma oncogene homolog B (avian) | 0.33 | 2.8E-09 |
| 231688_at | *MMP8* | matrix metallopeptidase 8 (neutrophil collagenase) | 0.34 | 2.6E-08 |
| 207802_at | *CRISP3* | cysteine-rich secretory protein 3 | 0.34 | 9.9E-09 |
| 206676_at | *CEACAM8* | carcinoembryonic antigen-related cell adhesion molecule 8 | 0.34 | 1.3E-07 |
| 207857_at | *LILRA2* | leukocyte immunoglobulin-like receptor, subfamily A (with TM domain), member 2 | 0.34 | 5.8E-22 |
| 203645_s_at | *CD163* | CD163 molecule | 0.36 | 8.0E-13 |
| 204834_at | *FGL2* | fibrinogen-like 2 | 0.36 | 2.4E-12 |
| 205119_s_at | *FPR1* | formyl peptide receptor 1 | 0.36 | 6.4E-15 |
| 201393_s_at | *IGF2R* | insulin-like growth factor 2 receptor | 0.36 | 1.1E-22 |
| 205936_s_at | *HK3* | hexokinase 3 (white cell) | 0.36 | 3.0E-21 |
| 225207_at | *PDK4* | pyruvate dehydrogenase kinase, isozyme 4 | 0.37 | 9.0E-13 |
| 212830_at | *MEGF9* | multiple EGF-like-domains 9 | 0.37 | 6.1E-23 |
| 212531_at | *LCN2* | lipocalin 2 | 0.37 | 1.7E-09 |
| 206177_s_at | *ARG1* | arginase, liver | 0.37 | 6.9E-09 |
| 204057_at | *IRF8* | interferon regulatory factor 8 | 0.37 | 4.9E-15 |
| 207674_at | *FCAR* | Fc fragment of IgA, receptor for | 0.37 | 5.1E-12 |
| 203936_s_at | *MMP9* | matrix metallopeptidase 9 (gelatinase B, 92kDa gelatinase, 92kDa type IV collagenase) | 0.37 | 9.9E-12 |
| 234987_at | *SAMHD1* | mitochondrial ribosomal protein 63 | 0.37 | 4.1E-09 |
| 212657_s_at | *IL1RN* | interleukin 1 receptor antagonist | 0.37 | 5.0E-14 |
| 204007_at | *FCGR3B* | Fc fragment of IgG, low affinity IIIb, receptor (CD16b) | 0.37 | 3.7E-15 |
| 205786_s_at | *ITGAM* | integrin, alpha M (complement component 3 receptor 3 subunit) | 0.37 | 2.9E-18 |
| 217911_s_at | *BAG3* | BCL2-associated athanogene 3 | 0.38 | 1.3E-13 |
| 203923_s_at | *CYBB* | cytochrome b-245, beta polypeptide | 0.38 | 1.2E-12 |
| 205174_s_at | *QPCT* | glutaminyl-peptide cyclotransferase | 0.38 | 4.2E-11 |

Figure S1. Differential gene expression in shared probe sets among Netherlands and Germany high-*WT1* gene sets. The differences in expression levels were significantly correlated among two gene sets (r^2^ = 0.81, *P* < 10^-18^).


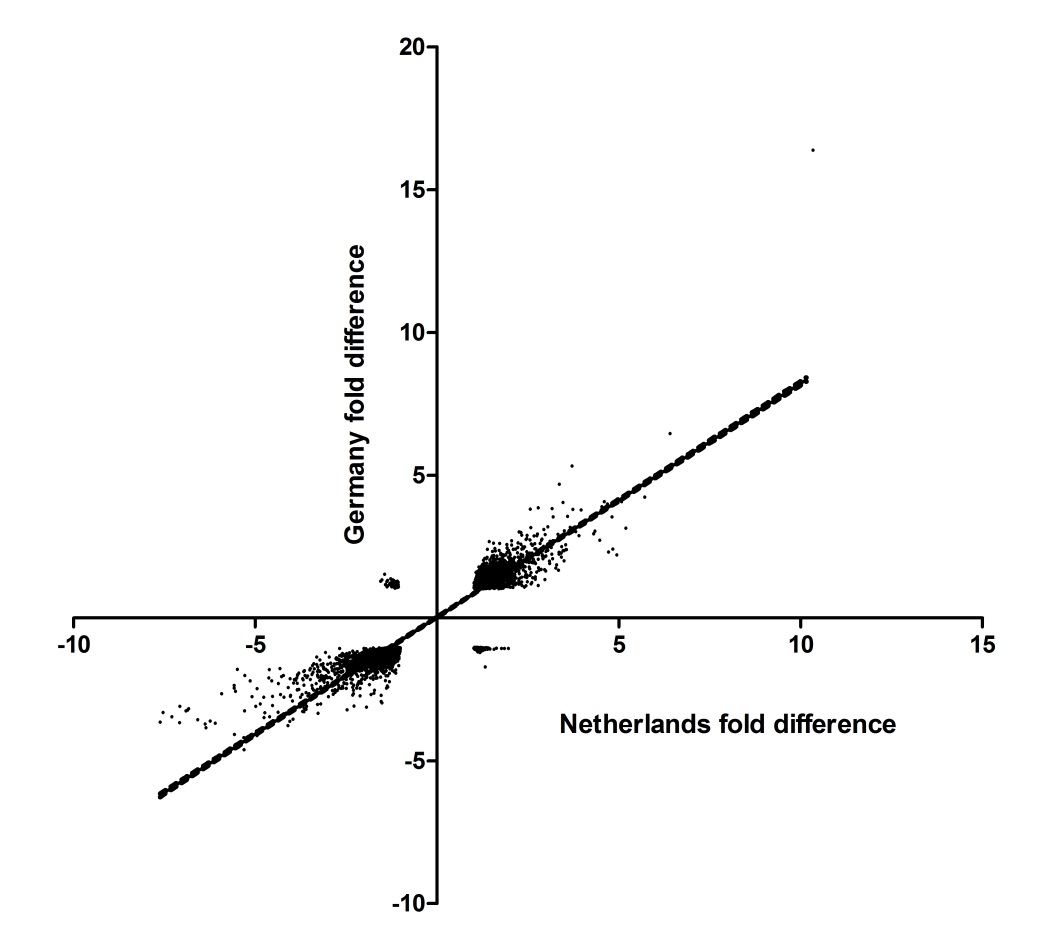


Figure S2. Univariate survival analysis of the supervised clustering by candidate lists of probe sets in order to obtain the optimal survival predictor. All candidate lists of 10 to 50 probe-sets from Netherlands gene expression study (S10 to S50) were used to classify the corresponding patients for overall survival (OS) and event-free survival (EFS). Hazard ratios (HRs; *top*) and statistical significance (*bottom*) for univariate Cox analysis of the OS and EFS. The S17 gene signature, comprising 17 probe-sets/ 16 unique gene transcripts, showed the highest overall correlation to both OS and EFS in terms of hazard ratio (HR) and statistical significance.


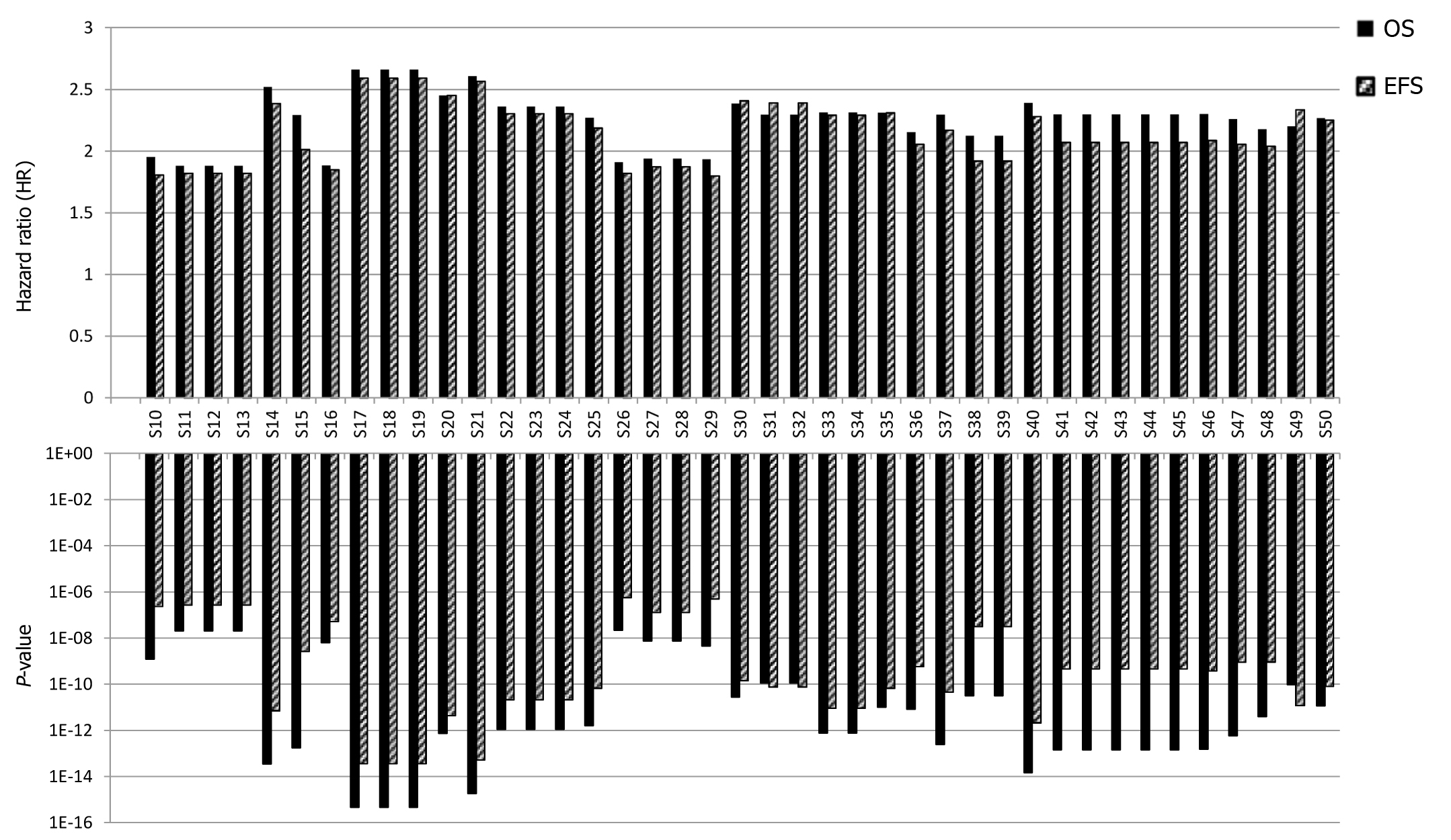


Table S3. The S17 signature (consisting of 16 unique gene transcripts) which optimally classified the high-WT1 cluster associated with poor prognosis in Netherlands high-*WT1* state. Two different probe-sets represented  *ADGRG1* expression level in the signature.

* This probe set was not present in the Germany GEP.

| Probe set | Gene Symbol | Gene Title | Netherlands fold dif. | FDR | Germany fold dif. | FDR |
| --- | --- | --- | --- | --- | --- | --- |
| 212070_at | *ADGRG1* | adhesion G protein-coupled receptor G1 | 3.54 | 2.4E-20 | 2.39 | 4.8E-16 |
| 220377_at | *KIAA0125* | FAM30A | 3.49 | 1.1E-19 | 2.00 | 2.7E-12 |
| 217963_s_at | *NGFRAP1* | nerve growth factor receptor (TNFRSF16) associated protein 1 | 3.34 | 4.0E-13 | 1.68 | 3.2E-05 |
| 217975_at | *WBP5* | WW domain binding protein 5 | 2.79 | 7.6E-20 | 2.23 | 3.2E-10 |
| 206582_s_at | *ADGRG1* | adhesion G protein-coupled receptor G1 | 2.45 | 2.1E-19 | 1.13 | 2.7E-04 |
| 217184_s_at | *LTK* | leukocyte receptor tyrosine kinase | 1.73 | 7.8E-10 | 1.12 | 1.4E-02 |
| 206574_s_at | *PTP4A3* | protein tyrosine phosphatase type IVA, member 3 | 1.59 | 9.6E-07 | 1.26 | 6.0E-04 |
| 226545_at | *CD109* | CD109 molecule | 1.49 | 3.2E-04 | 1.49 | 7.3E-03 |
| 231899_at | *ZC3H12C* | zinc finger CCCH-type containing 12C | 1.47 | 2.7E-06 | 1.34 | 5.2E-03 |
| 201481_s_at | *PYGB* | phosphorylase, glycogen; brain | 1.33 | 3.5E-04 | 1.13 | 3.0E-04 |
| 228345_at | *CHIC1* | cysteine-rich hydrophobic domain 1 | 1.24 | 5.7E-05 | 1.24 | 8.1E-06 |
| 1555628_a_at | *HAVCR2* | hepatitis A virus cellular receptor 2 | 0.74 | 1.6E-06 | -^*^ | -^*^ |
| 230689_at | *TMEM110* | transmembrane protein 110 | 0.72 | 1.1E-08 | 0.86 | 7.7E-08 |
| 217521_at | *HAL* | histidine ammonia-lyase | 0.67 | 7.8E-05 | 0.82 | 1.3E-02 |
| 204225_at | *HDAC4* | histone deacetylase 4 | 0.64 | 2.0E-13 | 0.88 | 5.7E-02 |
| 203771_s_at | *BLVRA* | biliverdin reductase A | 0.62 | 1.3E-17 | 0.59 | 2.0E-12 |
| 206277_at | *P2RY2* | purinergic receptor P2Y, G-protein coupled, 2 | 0.61 | 6.4E-09 | 0.63 | 3.7E-07 |

Table S4. Univariate analysis of the OS and EFS in the Netherlands AML series using Cox Regression analysis of the basic covariates and cytogenetic and molecular aberrations.

|  | OS | | | | EFS | | | |
| --- | --- | --- | --- | --- | --- | --- | --- | --- |
| Variable | *P*-val | HR | 95% CI for HR | | *P*-val | HR | 95% CI for HR | |
|  |  |  | Lower | Upper |  |  | Lower | Upper |
| Male gender | .006 | 1.258 | 1.070 | 1.481 | .008 | 1.255 | 1.062 | 1.482 |
| Age | .002 | 1.165 | 1.056 | 1.286 | .053 | 1.103 | .999 | 1.219 |
| Del(8) | .522 | 1.168 | .726 | 1.877 | .626 | 1.129 | .693 | 1.841 |
| Abn 11q23 | .473 | 1.246 | .683 | 2.274 | .849 | 1.063 | .566 | 1.997 |
| del(9q) | .196 | .399 | .099 | 1.604 | .150 | .360 | .090 | 1.447 |
| Complex karyot. | <.001 | 2.214 | 1.459 | 3.359 | .001 | 2.106 | 1.336 | 3.318 |
| inv(16) | .008 | .526 | .326 | .846 | .017 | .542 | .328 | .897 |
| del(5q)/(7q) | <.001 | 2.100 | 1.424 | 3.097 | <.001 | 2.561 | 1.720 | 3.812 |
| t(15;17) | .144 | .639 | .350 | 1.166 | .205 | .677 | .371 | 1.237 |
| t(8;21) | .002 | .447 | .271 | .740 | .002 | .427 | .250 | .731 |
| t(6;9) | .204 | 1.896 | .707 | 5.084 | .720 | 1.231 | .395 | 3.838 |
| t(9;22) | .229 | .049 | .000 | 6.684 | .336 | 1.748 | .560 | 5.454 |
| Abn 3q | .170 | 1.554 | .828 | 2.916 | .037 | 1.959 | 1.042 | 3.684 |
| *NPM1*-mut | .293 | .881 | .696 | 1.115 | .089 | .810 | .635 | 1.033 |
| *FLT3*-ITD | <.001 | 1.564 | 1.242 | 1.969 | .001 | 1.511 | 1.190 | 1.918 |
| *CEBPA* dbl. mut | .005 | .407 | .217 | .764 | .012 | .427 | .220 | .829 |
| *FLT3*-TKD | .025 | .621 | .410 | .941 | .012 | .565 | .363 | .880 |
| *NRAS*-mut | .283 | .809 | .550 | 1.191 | .932 | 1.017 | .686 | 1.509 |
| *KRAS*-mut | .563 | 1.399 | .449 | 4.361 | .689 | 1.261 | .404 | 3.934 |
| *IDH1*-mut | .454 | .845 | .543 | 1.314 | .831 | 1.045 | .699 | 1.560 |
| *IDH2*-mut | .289 | .792 | .514 | 1.219 | .522 | .871 | .569 | 1.331 |
| *WT1*-mut | .460 | 1.192 | .748 | 1.900 | .648 | 1.121 | .685 | 1.835 |
| S17 signature | <.001 | 2.718 | 2.147 | 3.442 | <.001 | 2.692 | 2.104 | 3.444 |

Table S5. Univariate analysis of the OS and RFS in Germany AML series using Cox Regression analysis of all potentially implicated variables.

|  | OS | | | | RFS | | | |
| --- | --- | --- | --- | --- | --- | --- | --- | --- |
| Variable | *P*-val | HR | 95% CI for HR | | *P*-val | HR | 95% CI for HR | |
|  |  |  | Lower | Upper |  |  | Lower | Upper |
| Age | <.001 | 1.354 | 1.263 | 1.452 | <.001 | 1.284 | 1.168 | 1.412 |
| Male gender | .010 | 1.293 | 1.063 | 1.572 | .323 | 1.146 | .874 | 1.503 |
| ECOG | <.001 | 1.320 | 1.177 | 1.481 | .023 | 1.210 | 1.027 | 1.425 |
| WBC | .030 | 1.016 | 1.002 | 1.031 | .174 | 1.016 | 0.993 | 1.039 |
| Hemoglobin | .946 | 1.003 | .916 | 1.099 | .983 | 1.001 | .884 | 1.135 |
| LDH | <.001 | 1.121 | 1.053 | 1.193 | .209 | 1.071 | 0.962 | 1.193 |
| Platelet | .220 | .966 | .915 | 1.021 | .347 | 1.033 | .966 | 1.104 |
| S17 signature | <.001 | 2.111 | 1.721 | 2.591 | <.001 | 2.635 | 1.965 | 3.532 |
| ELN2* | .016 | 1.324 | 1.054 | 1.662 | <.001 | 1.748 | 1.288 | 2.371 |
| ELN3* | .061 | 1.257 | .990 | 1.597 | .370 | 1.168 | .832 | 1.641 |
| ELN4* | <.001 | 2.057 | 1.643 | 2.577 | <.001 | 3.887 | 2.715 | 5.564 |

* The ELN2, ELN3, and ELN4 indicate the corresponding genetic ELN groups compared to the ELN1 group, respectively.

Table S6. Correlation of the individual S17 probe sets to the EFS in the Netherlands series using ROC curve analysis. Those probe-sets with AUCs (area under the curve) significantly above 0.5 correlate positively with the EFS, while those with AUCs less than 0.5 correlate negatively.

| Probe-set | Gene | Area under the curve | *P*-value | 95% CI,  lower limit | 95% CI,  upper limit |
| --- | --- | --- | --- | --- | --- |
| 226545_at | *CD109* | .675 | 9.7E-10 | .626 | .725 |
| 220377_at | *KIAA0125* | .668 | 4.7E-09 | .619 | .717 |
| 217963_s_at | *NGFRAP1* | .667 | 5.8E-09 | .616 | .718 |
| 231899_at | *ZC3H12C* | .654 | 7.7E-08 | .604 | .704 |
| 212070_at | *ADGRG1* | .647 | 3.0E-07 | .597 | .697 |
| 217521_at | *HAL* | .355 | 3.9E-07 | .300 | .409 |
| 217975_at | *WBP5* | .645 | 4.2E-07 | .594 | .696 |
| 206582_s_at | *ADGRG1* | .643 | 6.5E-07 | .592 | .693 |
| 204225_at | *HDAC4* | .358 | 6.7E-07 | .303 | .412 |
| 206574_s_at | *PTP4A3* | .638 | 1.6E-06 | .584 | .691 |
| 228345_at | *CHIC1* | .633 | 3.6E-06 | .580 | .686 |
| 230689_at | *TMEM110* | .377 | 1.9E-05 | .323 | .432 |
| 201481_s_at | *PYGB* | .402 | 6.6E-04 | .346 | .459 |
| 206277_at | *P2RY2* | .408 | 1.3E-03 | .351 | .464 |
| 217184_s_at | *LTK* | .414 | 2.6E-03 | .359 | .469 |
| 203771_s_at | *BLVRA* | .415 | 3.2E-03 | .360 | .471 |
| 1555628_a_at | *HAVCR2* | .417 | 3.6E-03 | .359 | .474 |

Table S7. Correlation of the gene scores obtained from cumulative gene expression of the *n* most significant probe-sets of the S17 to the EFS in the Netherlands series using ROC curve analysis. Both the AUC (area under the curve) and the *P*-value did change little after W4.

| Score | Area under the curve | *P*-value | 95% CI lower limit | 95% CI upper limit |
| --- | --- | --- | --- | --- |
| W2 | .694 | 1.4E-11 | .646 | .741 |
| W3 | .712 | 1.4E-13 | .665 | .759 |
| W4 | .717 | 4.2E-14 | .670 | .763 |
| W5 | .711 | 1.9E-13 | .665 | .757 |
| W6 | .718 | 2.8E-14 | .673 | .764 |
| W7 | .716 | 4.4E-14 | .671 | .762 |
| W8 | .714 | 8.5E-14 | .668 | .760 |
| W9 | .715 | 6.7E-14 | .669 | .761 |
| W10 | .718 | 2.8E-14 | .672 | .764 |
| W11 | .717 | 4.0E-14 | .671 | .763 |
| W12 | .720 | 1.6E-14 | .674 | .766 |
| W13 | .725 | 4.8E-15 | .679 | .770 |
| W14 | .722 | 1.1E-14 | .676 | .768 |
| W15 | .727 | 2.8E-15 | .681 | .772 |
| W16 | .727 | 2.3E-15 | .681 | .773 |
| W17 | .728 | 1.7E-15 | .683 | .774 |

Table S8. Multivariate analysis of the OS and EFS in the Netherlands AML series using Cox Regression analysis of those variables which were significant at the level of *P* < 0.1 in univariate Cox Regression.

|  | OS | | | | EFS | | | |
| --- | --- | --- | --- | --- | --- | --- | --- | --- |
| Variable | *P*-val | HR | 95% CI for HR | | *P*-val | HR | 95% CI for HR | |
|  |  |  | Lower | Upper |  |  | Lower | Upper |
| W4 score | <.001 | 1.660 | 1.275 | 2.162 | .002 | 1.540 | 1.175 | 2.018 |
| Age | .010 | 1.141 | 1.032 | 1.262 | .072 | 1.098 | .992 | 1.215 |
| Complex karyotype | .001 | 2.130 | 1.343 | 3.378 | .005 | 2.071 | 1.252 | 3.425 |
| inv16 | .035 | .554 | .319 | .960 | .074 | .605 | .348 | 1.051 |
| del5_7q | .031 | 1.669 | 1.048 | 2.659 | <.001 | 2.451 | 1.513 | 3.972 |
| t8_21 | .043 | .539 | .296 | .982 | .020 | .479 | .257 | .890 |
| *FLT3_ITD* | .001 | 1.631 | 1.229 | 2.166 | <.001 | 1.725 | 1.278 | 2.329 |
| *NPM1* | <.001 | .577 | .428 | .778 | <.001 | .524 | .384 | .715 |
| *CEBPA*-dbl-mut. | .012 | .416 | .210 | .826 | .010 | .405 | .204 | .805 |

Table S9. Multivariate analysis of the OS and RFS in Germany AML series using Cox Regression analysis of the ELN genetic groups and those variables which were significant at the level of *P* < 0.1 in univariate Cox Regression.

|  | OS | | | | RFS | | | |
| --- | --- | --- | --- | --- | --- | --- | --- | --- |
| Variable | *P*-val | HR | 95% CI for HR | | *P*-val | HR | 95% CI for HR | |
|  |  |  | Lower | Upper |  |  | Lower | Upper |
| W4 score | .003 | 1.404 | 1.119 | 1.761 | <.001 | 1.962 | 1.435 | 2.681 |
| Age | <.001 | 1.289 | 1.196 | 1.389 | <.001 | 1.200 | 1.083 | 1.330 |
| ELN2* | <.001 | 2.350 | 1.705 | 3.241 | <.001 | 3.149 | 2.124 | 4.668 |
| ELN3* | <.001 | 2.243 | 1.610 | 3.125 | .009 | 1.805 | 1.160 | 2.808 |
| ELN4* | <.001 | 3.333 | 2.388 | 4.654 | <.001 | 6.425 | 4.045 | 10.206 |

* The ELN2, ELN3, and ELN4 indicate the corresponding genetic ELN groups compared to the ELN1 group, respectively.

Figure S3. Antigen Presentation by MHC Class II as the most significant MetaCore pathway deregulated in Netherlands 2-fold high-*WT1* set (826 probe sets). Those genes with an adjacent color bar showed differed expression in high-*WT1* state, with the height of the bar as the relative decrease (blue) or increase (red) in expression level of the gene. Most key genes showed decreased expression, and hence the entire pathway seems to be downregulated in high-*WT1*.

Green arrows, activation; red arrows, inhibition; gray arrows, unspecified effect; B, binding; C, cleavage; Tn, transport.


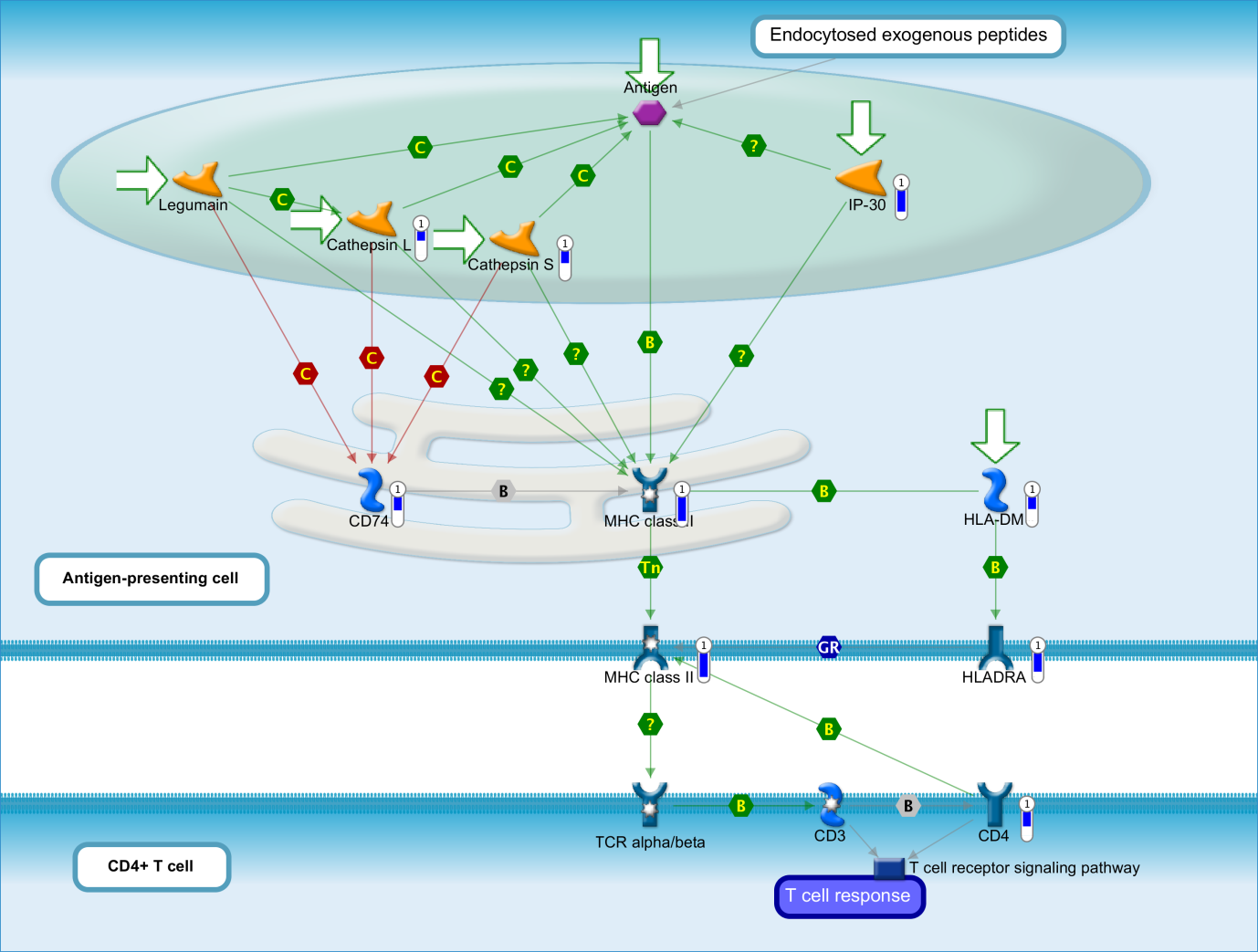


Figure S4. MetaCore Shortest Network as defined by the network formed among the S17 gene products and WT1, with not more than one connection. The S17 gene products are indicated using a red circle. One canonical pathway starts from WT1, goes through ESR1, PR, and P300, and ends to NFKB1 transcription factor. Other two canonical pathways start from IP3, go through Calmodulin, and terminate on either CREB1 or MEF2 transcription factor. The downregulated HDAC4 and upregulated WT1 act as two major hubs for the network.

Green arrows, activation; red arrows, inhibition; gray arrows, unspecified effect.


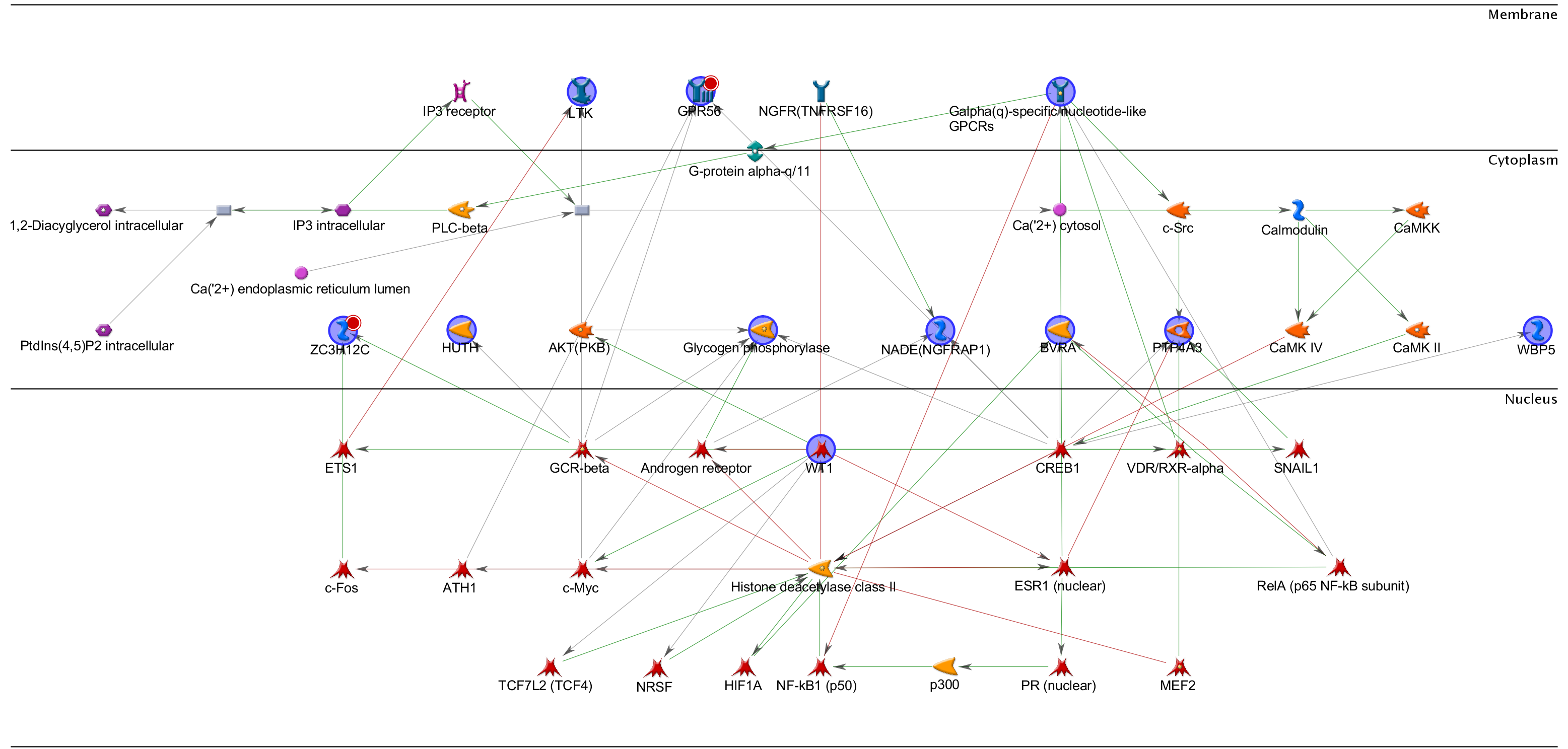

Supplement: Supplementary file 1 — Fig S1. Differential gene expression in shared probe sets among Netherlands and Germany high‐WT1 gene sets. The differences in expression levels were significantly correlated among two gene sets (r 2 = 0.81, P < 10−18). Fig S2. Univariate survival analysis of the supervised clustering by candidate lists of probe sets in order to obtain the optimal survival predictor. Fig S3. Antigen Presentation by MHC Class II as the most significant MetaCore pathway deregulated in Netherlands 2‐fold high‐WT1 set (826 probe sets). Those genes with an adjacent color bar showed differed expression in high‐WT1 state, with the height of the bar as the relative decrease (blue) or increase (red) in expression level of the gene. Most key genes showed decreased expression, and hence the entire pathway seems to be downregulated in high‐WT1. Fig S4. MetaCore Shortest Network as defined by the network formed among the S17 gene products and WT1, with not more than one connection. Table SI. Top 100 genes differentially expressed between the highest (Q4) and lowest (Q1) quartiles of WT1 expression level in the Netherlands series, or Netherlands high‐WT1 set. Table SII. Top 100 genes diffentially expressed between the highest (Q4) and lowest (Q1) quartiles of WT1 expression level in the Germany series, or Germany high‐WT1 set. Table SIII. The S17 signature (consisting of 16 unique gene transcripts) which optimally classified the high‐WT1 cluster associated with poor prognosis in Netherlands high‐WT1 state. Table SIV. Univariate analysis of the OS and EFS in the Netherlands AML series using Cox Regression analysis of the basic covariates and cytogenetic and molecular aberrations. Table SV. Univariate analysis of the OS and RFS in Germany AML series using Cox Regression analysis of all potentially implicated variables. Table SVI. Correlation of the individual S17 probe sets to the EFS in the Netherlands series using ROC curve analysis. Those probe‐sets with AUCs (area under the curve) significantly [file BJH-172-401-s001.docx]
